# Specific elimination of m.8993T>G mitochondrial haplotype in NARP cybrid cells by CRISPR-Cas9 system

**DOI:** 10.1038/s41598-026-49007-y

**Published:** 2026-04-28

**Authors:** Elvira Zakirova, Svetlana Sergeeva, Ksenia Morozova, Elena Kiseleva, Masashi Tanaka, Ilya Mazunin, Konstantin Orishchenko

**Affiliations:** 1 Federal Research Center Institute of Cytology and Genetics, Novosibirsk, 630090 Russia; 2 Novosibirsk State University, Novosibirsk, 630090 Russia; 3 University of Milano-Bicocca, Milan, 20126 Italy; 4 Department of Neurology, Juntendo University Graduate School of Medicine, Tokyo, 113- 8421 Japan; 5 The Center for Bio- and Medical Technologies, Moscow, 121205 Russia; 6 Department of Biology and Genetics, Petrovsky Medical University, Moscow, 119435 Russia

**Keywords:** CRISPR-SpCas9, Mitochondria, NARP syndrome, m.8993T>G point mutation, Heteroplasmy shift, Cell biology, Diseases, Genetics, Molecular biology, Neuroscience

## Abstract

Mutations in mitochondrial DNA can cause a wide range of neuromuscular and neurodegenerative diseases in humans. The heteroplasmy level, coexistence of both wild-type and mutant mtDNA within a cell, determines the manifestation and severity of disease symptoms. Therefore, the development of strategies to shift heteroplasmy toward wild-type mtDNA is critical for advancing therapies for mitochondrial disorders. Mitochondrial localization of the modified CRISPR-Cas9 system components was analyzed using western blotting, immunocytochemical staining, confocal microscopy, and immunoelectron microscopy. To assess the heteroplasmy shift, cybrid cell lines carrying the clinically relevant m.8993T>G variant associated with Neuropathy, Ataxia, and Retinitis Pigmentosa (NARP) syndrome were used. The heteroplasmy level was evaluated by RFLP analysis of PCR-amplified mtDNA fragments encompassing the mutation site. We adapted the widely used CRISPR-Cas9 system, originally designed for editing nuclear DNA, to induce a heteroplasmy shift of the pathogenic m.8993T>G point mutation in human cybrid cells. We demonstrated that the modified components of the CRISPR-Cas9 system – the mitoCas9 nuclease fused to the mitochondrial targeting sequence from COX8A, and a single guide RNA containing a mitochondrial import determinant within the tetraloop – are effectively imported into the mitochondrial matrix. Transient transfection of the modified sgRNA into cybrid cells stably expressing mitoCas9 resulted in a detectable shift in heteroplasmy. Mitochondria-targeted CRISPR-SpCas9 system can selectively reduce the mutant m.8993T>G mtDNA load in human cybrid cells, achieving a reproducible shift in heteroplasmy.

**Supplementary Information:**

The online version contains supplementary material available at 10.1038/s41598-026-49007-y.

## Introduction

Mitochondria are essential organelles responsible for cellular energy production through oxidative phosphorylation. Each mitochondrion contains its own genome, mitochondrial DNA (mtDNA), which encodes key components of the respiratory chain complexes^1^. Mutations in mtDNA can impair mitochondrial function, leading to a spectrum of neuromuscular and neurodegenerative disorders^2^. Unlike nuclear DNA, mtDNA is present in multiple copies within each cell, typically resulting in heteroplasmy, where mutant and wild-type mtDNA coexist. The ratio between mutant and wild-type mtDNA determines the clinical severity of mitochondrial diseases, with even moderate reductions in mutant load capable of significantly alleviating disease symptoms^3^.

A well-characterized pathogenic mutation, m.8993T>G in the mitochondrial *MT-ATP6* gene, is causally associated with Neuropathy, Ataxia, and Retinitis Pigmentosa syndrome (NARP, OMIM 551500), a severe progressive neurodegenerative disease. Therapeutic strategies aimed at treating mitochondrial diseases often focus on shifting mtDNA heteroplasmy in favour of wild-type genomes, thereby reducing the pathogenic mtDNA fraction below a clinical threshold. Initial advancements employing mitochondria-targeted nucleases, such as restriction enzymes, engineered meganucleases, mitochondrial zinc-finger nucleases (mitoZFs), and mitochondrial transcription activator-like effector nucleases (mito-TALENs), have demonstrated promising outcomes^4,5^. In particular, tools targeting the m.8993T>G mutation have been developed using restriction enzymes (e.g., SmaI^6^ and XmaI^7^) and mitoZFs, with substantial contributions from Minczuk’s group^8–12^. However, these approaches, particularly mitoZFs, face challenges including cellular toxicity, off-target cleavage, and complex engineering and delivery requirements.

The emergence of the CRISPR-Cas system, renowned for its simplicity, versatility, and specificity, has revolutionized nuclear genome editing^13^. However, adapting CRISPR-Cas technology for mitochondrial genome editing requires targeted modifications to both the Cas nuclease and the single guide RNA (sgRNA). To direct Cas nucleases to mitochondria, mitochondrial targeting sequences (MTS), such as COX8A, have been successfully employed, often combined with mitochondrial-specific 3’-untranslated regions (3’-UTRs), such as from the *SOD2* gene, facilitating mitochondrial RNA import and translation^14,15^. In contrast, the mitochondrial targeting of sgRNAs remains a major challenge. Several RNA hairpin structures have been identified as mitochondrial RNA import determinants, including domains from human 5S rRNA^16^, yeast lysine tRNA (F- and D-stems)^17^, and RNAs H1 and MRP^18^. These structures have been fused to either 5’ or 3’ - ends or integrated internally - within gRNAs to enhance mitochondrial import without significantly affecting in vitro nuclease activity^19^. Notably, recent findings indicate that unmodified crRNAs for AsCas12a are intrinsically capable of mitochondrial import, potentially due to inherent secondary structures^20^.

To date, evidence for CRISPR-Cas system activity within mitochondria primarily arises from observations of reduced mtDNA copy number, indicative of mtDNA cleavage followed by degradation. In mammalian cells mitochondria preferentially degrade damaged DNA rather than repair double-strand breaks, thereby supporting the therapeutic strategies based on targeted cleavage to selectively eliminate mutant mtDNA^21^.

In the current study, we report the first application of an optimized CRISPR-SpCas9 system specifically engineered to eliminate the m.8993T>G mitochondrial haplotype in NARP cybrid cells. Mitochondrial localization of SpCas9 was rigorously validated through immunocytochemistry, protein blotting, and electron microscopy. In parallel, confocal microscopy confirmed mitochondrial localization of the engineered sgRNAs. Notably, our CRISPR-SpCas9 approach demonstrated a consistent and reproducible 16–20% reduction in mutant mtDNA heteroplasmy levels in targeted cytoplasmic hybrid cell lines with stable expression of mitoCas9 nuclease. These results highlight the considerable therapeutic potential of CRISPR-SpCas9 system for the precise correction of pathogenic mitochondrial genome mutations.

## Materials and methods

### Plasmids and transfection

The gene encoding the mitoCas9 nuclease was amplified from the pMitoCas9 plasmid, constructed in our previous work^15^, using primers containing SfiI restriction sites at their 5′ ends. The resulting fragment was cloned into the pSBbi-GP vector (AddGene #60511^22^, via SfiI digestion and ligation, generating the pSBbi-GP-mitoCas9 vector. For stable integration of mitoCas9 into the genome of cybrid cells using the Sleeping Beauty system, the pCMV(CAT)T7-SB100 plasmid (AddGene #34879^23^, encoding the SB100X transposase, was used. Plasmids for sgRNA expression containing mitochondrial import determinants were prepared as previously described in our work^19^. The sgRNA spacer sequence was replaced with a sequence corresponding to positions 8972–8991 of the mtDNA rCRS using oligonucleotides with complementary ends via the Gibson assembly method. pTurboGFP-mito (Evrogen) and pTagRFP-mito (Evrogen) were used for mitochondrial labeling. pEGFP-N1 (Clontech) was used to label the cytoplasm. Plasmid sequences are available upon request. Transient transfection was performed using Lipofectamine 3000 (Invitrogen) and OptiMEM Reduced Serum-Medium (Gibco), following the manufacturer’s protocols.

### Cell lines and cell culture

Cybrid cell lines NARP3-1 and NARP3-2, carrying the m.8993T>G mutation in the *MT-ATP6* gene with heteroplasmy levels of approximately 95% and 50%, respectively, were established in previous work^6^. The mitoCas9 gene was introduced into cybrid cell lines nuclear genome using the Sleeping Beauty system^22^. Cybrid cell lines were transfected in a six-well plate with a 1:10 ratio of pCMV(CAT)T7-SB100 plasmid and pSBbi-GP-mitoCas9 using Lipofectamine 3000 transfection reagent. After two days, cells were subjected to selection by adding puromycin (Gibco) to the culture medium at a concentration of 1 µg/mL. Сells were allowed to propagate in selective media for 7 days. 10^3^ cells were seeded into 10 cm cell culture dishes, and after colony formation, individual colonies were selected using the automated cell and colony picking system AVISO CellCelector (ALS). Cybrid cell line 2SA with wild-type mtDNA was established in previous work^6^. All cell lines were maintained in high glucose DMEM (Gibco) supplemented with 10% FBS (Invitrogen), a mixture of antibiotics penicillin and streptomycin at a concentration of 100 µg/mL each (Invitrogen), 0.11 mg/mL sodium pyruvate (Sigma), and 0.1 mg/mL uridine (Sigma) at 37 °C in 5% CO_2_. Prior to each experiment, cultures were screened for mycoplasma contamination using real-time PCR with the BioMaster Myco-visor mix (Biolabmix). Total DNA, was extracted using the ExtractDNA Blood & Cells kit (Evrogen) and served as the template for downstream analyses.

### Evaluation of heteroplasmy

The heteroplasmy level of the m.8993T>G mutation in the *MT-ATP6* gene was determined by RFLP analysis of PCR-amplified mtDNA fragments encompassing the mutation site. Total genomic DNA was extracted from cells as above. PCR amplicons were generated using the primers listed in Supplemental Table 2. Amplicons were generated in a reaction containing 1× Q5 Reaction Buffer (NEB), 12.5 pmol of each primer, 0.2 mM of dNTPs, 0.5U of Q5^®^ High-Fidelity DNA Polymerase (NEB) in a final volume of 25 µl. Thermal cycling conditions were as follows: initial denaturation at 98 °C for 30 s, 35 cycles of 98 °C for 10 s, 60 °C for 20 s, 72 °C for 1 min; followed by a final extension at 72 °C for 2 min and hold at 12 °C. The resulting PCR products were digested with 1 µl AvaI (10U) (NEB) in CutSmart Buffer overnight at 37 °C. Digested products were resolved on a 1.2% agarose gel. AvaI generates two fragments (960 and 718 bp for the PCR product amplified with H9931/L8305 primers, and 960 and 345 bp for the product amplified with H9931/P24 primers) in the presence of mutant mtDNA. In contrast, wild-type mtDNA yields a single fragment of 1678 bp–1305 bp when amplified with H9931/L8305 or H9931/P24 primers, respectively. As a negative control, mtDNA isolated from the 2SA cell line with (homoplasmic WT mtDNA) was used. Quantification of digested PCR products was performed by gel densitometry using Image Lab software (version 3.0, build 11, Bio-Rad).

### Subcellular fractionation and western blotting

Cytoplasmic (C) and mitochondrial (M) protein fractions were isolated using the QProteome Mitochondria Isolation Kit (Qiagen), according to the manufacturer’s instructions. Total cellular protein (T) was extracted from 5 × 10⁶ cells lysed in RIPA buffer (50 mM Tris–HCl, pH 7.6; 150 mM NaCl; 1% Triton X-100; 0.5% sodium deoxycholate; 0.1% SDS) supplemented with Pierce Protease and Phosphatase Inhibitor Mini Tablets, EDTA-free (Thermo Fisher Scientific). Lysates were incubated on ice for 30 min and sonicated using a Sonopuls instrument (Bandelin) under the following conditions: three 10-second cycles at 30% power, with 60-second intervals. Homogenates were clarified by centrifugation at 14 000 × g for 20 min at 4 °C, and supernatants collected as soluble protein fractions. Protein concentration was measured using the Pierce BCA Protein Assay Kit (Thermo Fisher Scientific). Equal amounts (15 µg) of protein, denatured in Laemmli buffer, were separated by 4–12% Bolt™ Bis-Tris Plus Mini Protein Gels (Invitrogen™) in Bolt™ MES SDS Running Buffer (Invitrogen™). Following electrophoresis, proteins were transferred to a PVDF membrane 0.2 μm pore size (Invitrogen™) by wet transfer in 1 × Bolt™ Transfer Buffer (Invitrogen™). Membranes were blocked in EveryBlot Blocking Buffer (Bio-Rad) for 5 min at room temperature (RT). Subsequently, membranes were incubated with primary antibodies diluted in EveryBlot Blocking Buffer: anti-DYKDDDDK Tag Monoclonal Antibody (FG4R) (1:500, MA1-91878, Thermo Fisher Scientific), anti-GAPDH (1:2500, MA5-15738, Thermo Fisher Scientific), and anti-TOM20 Rabbit mAb (1:5000, A19403, ABclonal), for 1 h at RT or overnight at 4 °C. This was followed by washing membrane 5 × 5 min with TBST buffer (Tris buffered saline with 0.05% Tween 20) and incubation with HRP-conjugated secondary antibodies, also diluted in EveryBlot Blocking Buffer (Bio-Rad), for 1 h at RT. Membranes were washed six times for 5 min each in TBST, incubated with SuperSignal™ West Pico PLUS Chemiluminescent Substrate (Thermo Scientific™), and visualized using the iBright™ FL1500 Imaging System (Invitrogen™).

### Immunofluorescence and microscopy

Cells were grown on coverslips, washed three times with phosphate-buffered saline (PBS) and fixed for 15 min at 37 °C in a 4% paraformaldehyde in PBS. Following fixation, coverslips were washed twice with PBS for 5 min each, and cells were permeabilized with 0.25% Triton X-100 in PBS for 10 min at RT. To block nonspecific antibody binding, cells were washed twice with PBS for 5 min each and incubated for 30 min at 37 °C in a 10% BSA in PBS. Cells were then washed three times with PBS and incubated with mouse monoclonal anti-FLAG M2 primary antibodies (1:500, F1804, Sigma-Aldrich) diluted in 3% BSA in PBS for 2 h at 37 °C. After two washes with PBS for 5 min each, cells were incubated with Alexa Fluor 488-conjugated anti-mouse IgG secondary antibodies (1:500, A-11001, Invitrogen) diluted in 3% BSA in PBS for 45 min at 37 °C. Cells were subsequently washed three times with PBS for 5 min each and mounted in ProLong Diamond Antifade Mountant with DAPI (Invitrogen).

Single guide RNAs incorporating mitochondrial import determinants were transcribed in vitro using the HiScribe T7 Quick High-Yield RNA Synthesis Kit (NEB) with the addition of Fluorescein-12-UTP (Sigma-Aldrich) according to the manufacturer’s instructions. Templates for in vitro transcription were amplified by PCR from sgRNA plasmids described previously using high-fidelity Q5 polymerase (NEB) and primers listed in Supplemental Table 2. The resulting RNA transcripts were purified by phenol-chloroform extraction followed by ethanol precipitation. Cells were grown using POC-R2 Cell Cultivation System (Pecon) on cover glasses and transiently transfected with 2.5 µg labeled sgRNA. After 48 h mitochondria were stained with 150 nM MitoTracker^®^ Red CMXRos dye (Invitrogen) for 30 min at 37 °C, according to the manufacturer’s protocol.

Images were acquired using an LSM 780 NLO laser scanning confocal microscope (Carl Zeiss) equipped with a ×63/1.4 oil immersion objective (Carl Zeiss). Image analysis was performed using ZEN 2010 software (version 6.0.0.320, Carl Zeiss).

The degree of co-localization between two targets was assessed by measuring the number of co-localized pixels obtained through sequential scanning in different fluorescence channels (red and green). The Pearson correlation coefficient (PCC) was calculated using ZEN 2010 software (version 6.0.0.320, Carl Zeiss). Threshold values were automatically determined for each image using the Costes method^24^. Co-localization analysis was performed on multiple cells and optical sections. PCC data are presented as the mean ± standard deviation (SD). Method validation for co-localization analysis was carried out using images obtained from a fluorescent microscope, sourced from a reference co-localization test from The Colocalization Benchmark Source (CBS) (www.colocalization-benchmark.com/).

### Transmission electron microscopy and immunoelectron microscopy

Cell preparation for transmission electron microscopy (TEM) was performed following a previously described procedure^25^. For immunoelectron microscopy, cells grown on Melinex film (Agar Scientific) were fixed in 4% paraformaldehyde with 0.25% glutaraldehyde in 0.1 M phosphate buffer for 3 h at RT. After fixation, cells were rinsed in the same buffer and dehydrated in increasing concentrations of ethanol (30%, 50%, 70%, 96%, 100%) for 10 min on ice and 30 min at −20 °C in isopropanol.

Subsequently, cells were gradually incubated in a mixture of isopropanol and LR Gold embedding medium (EMS) in different ratios: 1:3, 1:1, and 3:1 (v/v), each for 1 h at −20 °C. Embedding was performed using the LR Gold resin kit (EMS). The following day, cells were transferred into embedding moulds with fresh LR Gold medium containing 0.1% benzoyl peroxide. Sealed moulds were placed in a polymerization chamber under ultraviolet light. Polymerization occurred for 48 h at −20 °C, followed by gradual warming of the specimens to room temperature and further polymerization under ultraviolet light for an additional day at + 20 °C.

Semithin and ultrathin sections were cut using a diamond knife (Diatome) on an ultramicrotome UMC-7 (Leica). Immunolabeling on ultrathin sections with a thickness of 60–70 nm was carried out on nickel grids (EMS G200-Ni) using monoclonal anti-FLAG M2 primary antibodies (1:1000, F1804, Sigma), followed by secondary antibodies conjugated with 10 nm colloidal gold particles (1:50, ab39619, Abcam). The sections were examined and imaged using a JEOL-1400 transmission electron microscope (JEOL) equipped with a Veleta camera (Olympus) and iTEM software (version 5.1, Olympus).

Morphometric analysis was performed on ultrathin sections using iTEM software (version 5.1, Olympus). For each cell line, a total of 300 mitochondria were randomly selected from multiple sections and analyzed. The analysis was conducted using a blinded assessment approach, i.e., on coded samples.

### Droplet digital PCR

Primers and probes used for droplet digital PCR (ddPCR) are listed in Supplemental Table 2. The ddPCR analysis was performed using the QX100 system (BioRad). The reaction mixture was in a volume of 22 µL and comprised 11 µL of 2× ddPCR Supermix for Probes (BioRad), 1.1 µL of 20 × probe labeled with FAM/primers mixture (250/900 nM final concentrations), 1.1 µL of 20 × probe labeled with HEX/primers mixture (250/900 nM final concentrations), 4.4 µL of nuclease-free water and 4.4 µL of genomic DNA (10 ng/µL), pre-digested with HindIII. Thermal cycling was performed as follows: initial denaturation at 95 °C for 10 min, followed by 45 cycles of denaturation at 94 °C for 1 min and annealing/extension at 60 °C for 2 min, and a final extension step at 98 °C for 10 min. All samples were analyzed in triplicate. Data were processed using QuantaSoft software (version 1.7.4.0917, BioRad).

### MtDNA copy number evaluation

Total genomic DNA was extracted from cells as above. MtDNA copy number was quantified by real-time PCR using primers targeting the mitochondrial D-loop region and the single-copy nuclear gene Beta-2-Microglobulin (B2M) as a reference. The primers are listed in Supplemental Table 2. Each 25 µL reaction contained 1× BioMaster HS-qPCR SYBR Blue PCR mix (Biolabmix), 300 nM of each primer, and 25 ng of total DNA. Amplification was performed using a CFX96 Touch Real-Time PCR Detection System (BioRad) under standard cycling conditions. For each experimental condition, three independent biological replicates were analyzed, each with three technical replicates. The relative mtDNA copy number was calculated using the 2^−ΔΔCt^ method, comparing Ct values of D-loop and B2M, and normalized to untreated samples.

### Oxygen consumption analysis

Cybrid cells (~ 5,000 per well) were plated onto 8-well Seahorse XF HS Miniplates (Agilent) according to the manufacturer’s instructions. On the day of the assay, the culture medium was replaced with Seahorse XF DMEM Medium, pH 7.4 (Agilent), supplemented with 1 mM sodium pyruvate, 2 mM glutamine, and 10 mM glucose. Cells were incubated at 37 °C for 1 h prior to analysis. The Seahorse XF Mito Stress Test (Agilent) was performed by sequentially injecting 1.5 µM oligomycin, 1 µM FCCP, and 0.5 µM rotenone/antimycin A. Oxygen consumption rate (OCR) was measured using the Seahorse XF HS Mini Analyzer (Agilent). Following the assay, cells in each well were imaged using an Axio Observer.Z1 inverted microscope (Carl Zeiss) with a 5× objective. Cell numbers were quantified using ImageJ software (version 1.54p, https://imagej.net) for data normalization.

### Statistical methods

Normality of data distribution was assessed using the Shapiro–Wilk test. Morphometric analysis results were processed using R (version 4.5.2, https://cran.r-project.org). Differences in mitochondrial length and circularity distributions between experimental groups were evaluated using two-way analysis of variance (ANOVA) with replication, with cell line and mitoCas9 expression as factors. The significance threshold for the ANOVA test was selected considering the Bonferroni correction for multiple testing and was set to P-value < 0.05/4 = 0.0125, where 4 is the number of groups based on mitochondrial length and roundness coefficient. Pairwise comparisons between groups were evaluated using the post hoc Tukey test.

Statistical analysis was performed in R (version 4.5.2, https://cran.r-project.org). Because baseline heteroplasmy levels differed between parental and mitoCas9-expressing cell lines, statistical analyses were performed separately for each cell line and mitoCas9 condition. For each experimental background (NARP3-1, NARP3-1 mitoCas9, NARP3-2, and NARP3-2 mitoCas9), heteroplasmy levels were analyzed using two-way analysis of variance (ANOVA) with treatment and time point (day 2 and day 6) as factors. Post hoc pairwise comparisons were performed to evaluate differences between treatment groups and changes over time. Each experimental condition contained nine biological replicates.

Oxygen consumption rate and respiration parameters were analyzed with GraphPad Prism (version 8.0.1, https://www.graphpad.com) using the non-parametric Mann-Whitney test.

## Results

### Targeting SpCas9 nuclease to mitochondria does not affect level of heteroplasmy in cybrid cells

**Fig. 1:**
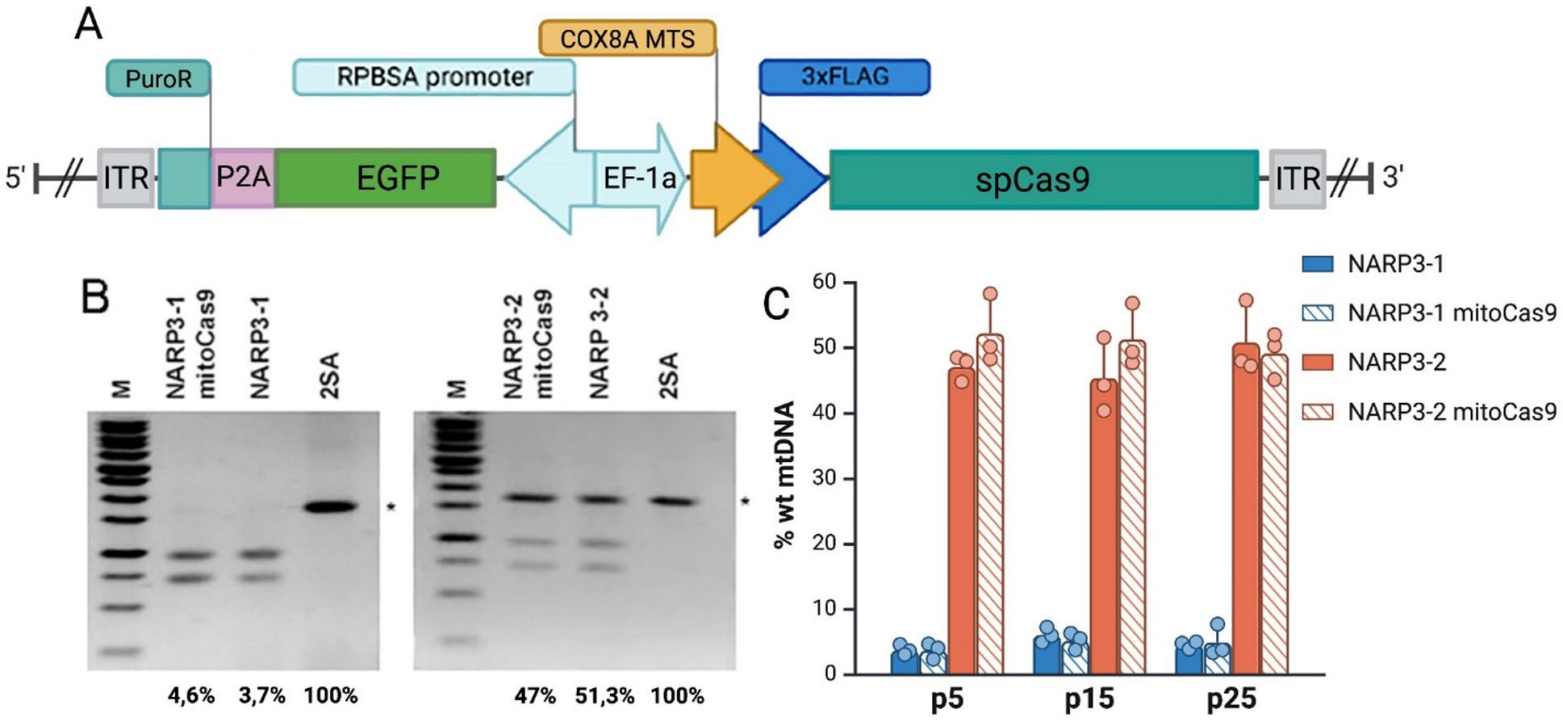
Design cybrid cell lines with stable mitoCas9 expression. (**A**) Schematic structure of the construct used for mitoCas9 genomic integration into cybrid cells by optimized Sleeping Beauty transposon system. Mitochondrial targeting of spCas9 is facilitated by a 23-amino acid MTS from cytochrome c oxidase subunit 8 A (COX8A MTS). SpCas9 - gene encoding the Cas9 nuclease from *S. pyogenes* with optimized codon usage for mammalian cell expression; 3xFLAG tag – synthetic polypeptide for protein detection; EF-1а – constitutive elongation factor 1 α promoter; RPBSA – synthetic promoter; EGFP – enhanced green fluorescent protein; P2A – porcine teschovirus-1 2A 19‐amino acid‐long peptide; PuroR – puromycin N-acetyltransferase confers resistance to puromycin; ITR - inverted terminal repeat required for transposition. (**B**) PCR-RFLP analysis of m.8993T>G heteroplasmy levels in parental and the transgenic clones NARP3-1 mitoCas9 and NARP3-2 mitoCas9. The percentage of wild-type mtDNA quantified by PCR-RFLP is shown below each corresponding electrophoresis lane. Mitochondrial DNA isolated from the 2SA cell line, which lacks the m.8993T>G mutation, was used as a negative control. * - uncut PCR product. M – Sky-High DNA marker For original gel images, please refer to supplemental materials (Fig. S1). (**С**) PCR-RFLP analysis of mtDNA heteroplasmy dynamics in parental and transgenic cell lines across passages 5, 15, 25, respectively. Data represent mean ± SD (*n* = 3 biological replicates). Statistical comparisons between parental and transgenic lines were performed using an unpaired two-tailed Student’s t-test; all comparisons were not significant (*p* > 0.05). For original gel images, please refer to supplemental materials (Fig. S2).

To import the SpCas9 nuclease - the protein component of the CRISPR-Cas9 system - into mitochondria, we used a common approach involving the fusion of a mitochondrial targeting sequence (MTS) to the N-terminus of the protein. Since transient transfection of plasmid DNA leads to variable expression levels of the target gene across cells and is generally inefficient in cybrid cell lines, we generated transgenic cybrid cell lines with stable expression of the mitochondrially targeted SpCas9 nuclease (mitoCas9). For this, we used an optimized Sleeping Beauty transposon system^22^. The gene encoding *Streptococcus pyogenes* Cas9, codon-optimized for expression in mammalian cells, was cloned into the pSBbi-GP plasmid along with the mitochondrial targeting sequence of cytochrome c oxidase subunit 8 A (COX8A MTS) at the N-terminus of SpCas9 (Fig. 1A). The resulting construct, pSBbi-GP-mitoCas9, enables constitutive expression of mitoCas9 in mammalian cells under the control of the constitutive EF-1α promoter. A 3xFLAG peptide was inserted between the COX8A MTS and SpCas9 for detection of the nuclease via immunofluorescence staining or western blot. The plasmid also contains elements necessary for nuclear genomic integration and selection of transgenic clones.

For stable genomic integration, we used NARP3-1 and NARP3-2 cybrid cell lines, which show heteroplasmy for the m.8993T>G mutation at approximately 95% and 50%, respectively^6^. Co-transfection of NARP3-1 and NARP3-2 cells with pSBbi-GP-mitoCas9 and pSB100X (encoding components of the Sleeping Beauty system), followed by selection in puromycin-containing medium, produced several EGFP+ subclones. From these, one EGFP+ subclone from each line (NARP3-1 mitoCas9 and NARP3-2 mitoCas9) was selected for further experiments based on the highest EGFP expression level, which indirectly indicates the number of mitoCas9 integrations in the cell genome. PCR-RFLP analysis of the m.8993T>G heteroplasmy level in the resulting transgenic lines showed that heteroplasmy remained at the levels observed in the parental NARP3-1 and NARP3-2 lines (Fig. 1B).

Considering previous reports suggesting that expression of certain CRISPR nucleases may affect mitochondrial health and potentially alter heteroplasmy levels^14^, we longitudinally monitored heteroplasmy in NARP3-1 mitoCas9 and NARP3-2 mitoCas9 cell lines over 25 passages of continuous cultivation. The m.8993T>G heteroplasmy level was measured at passages 5, 15, and 25, and it remained unchanged (Fig. 1C). Additionally, EGFP expression levels in NARP3-1 mitoCas9 and NARP3-2 mitoCas9 cell lines were characterized via flow cytometry, and the number of mitoCas9 transgene insertions into the genome was determined by droplet digital PCR. We found that NARP3-1 mitoCas9 harboured 25 transgene copies, while NARP3-2 mitoCas9 carried 14 copies, and these copy numbers correlated with EGFP fluorescence intensity (Supplementary Table 1). These findings suggest that m.8993T>G heteroplasmy levels remained stable across cell lines with varying transgene copy numbers during long-term cultivation, we conclude that mitoCas9 expression in cybrid cell lines does not lead to a shift in heteroplasmy.

### Modified SpCas9 localizes within the mitochondria of cybrid cells

**Fig. 2:**
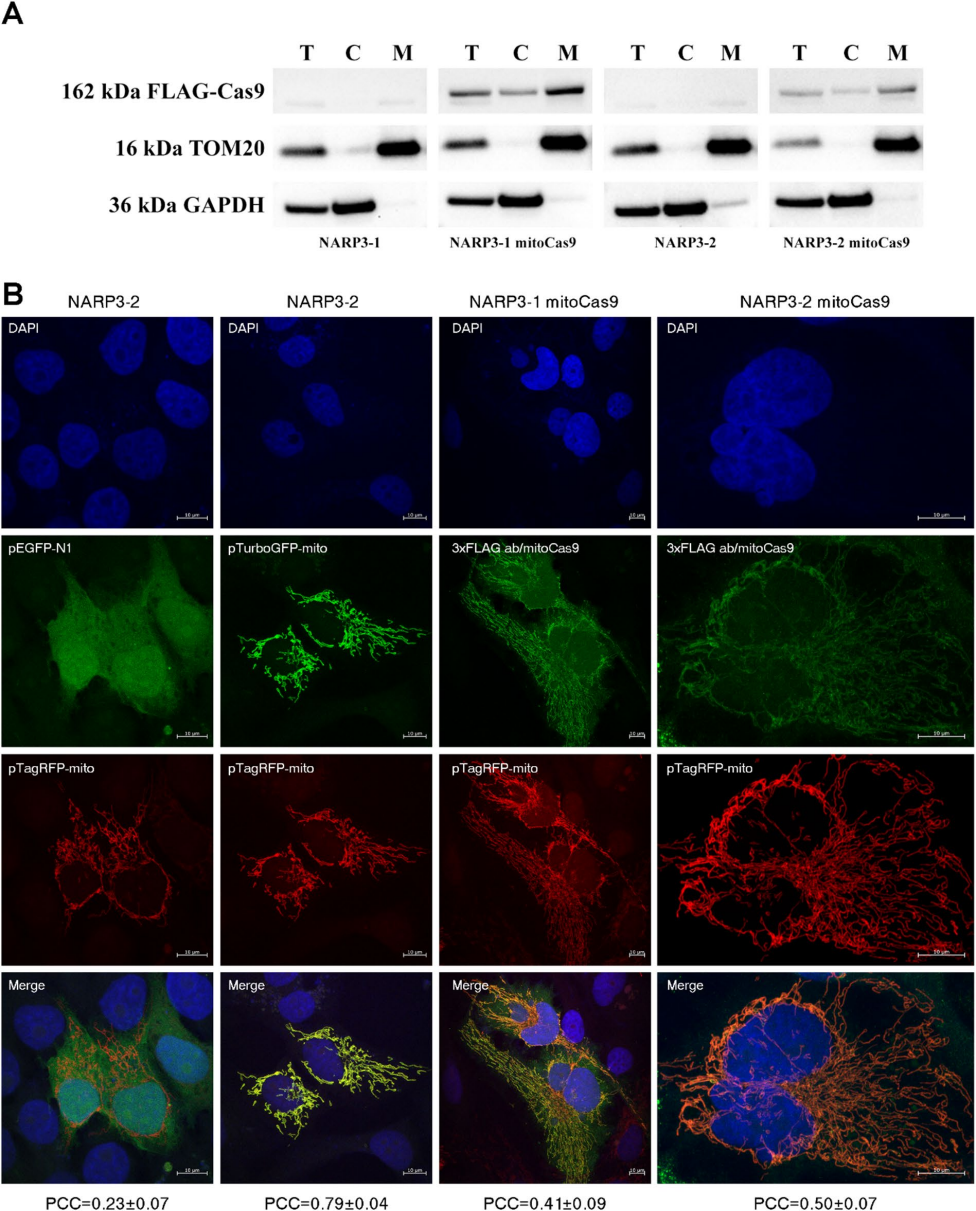
MitoCas9 expressed in the transgenic cybrid cell lines exhibits partial localization to mitochondria (**A**) Immunoblots of cellular fractions of parental and transgenic NARP cybrids. Cells were fractionated into cytoplasmic (C) and mitochondrial (M) protein fractions and along with total cell lysates (T), were analyzed by western blotting. The antibodies used are shown on the left: 3xFLAG (epitope tag fused to mitoCas9); TOM20 (outer mitochondrial membrane protein); GAPDH (predominantly cytoplasmic protein). For original images, please refer to supplemental materials (Fig. S3). (**B**) Immunofluorescent staining of the NARP transgenic cell lines. The mitoCas9 nuclease was labeled with 3xFLAG-tag/Alexa Fluor 488 antibodies (green signal), while mitochondria were labeled with expression of TagRFP with mitochondrial localization (red signal). DAPI was used for nuclear staining (blue signal). Quantitative assessment of mitoCas9 nuclease and mitochondrial colocalization was measured using the Pearson correlation coefficient (PCC) for green and red channel pixels. Data are presented as the mean ± SD (*n* = 3 independent experiments). Scale bar, 10 μm.

To evaluate the intracellular localization of mitoCas9, we performed subcellular fractionation to separate cytoplasmic (C) and mitochondrial (M) protein fractions. These subcellular fractions, along with total cell lysates (T), were analyzed by western blot using antibodies against the 3xFLAG synthetic peptide (to detect mitoCas9), TOM20 (outer mitochondrial membrane protein), and GAPDH (a predominantly cytoplasmic protein) (Fig. 2A). MitoCas9-specific bands were detected in subcellular fractions as well as in total lysates of NARP3-1 mitoCas9 and NARP3-2 mitoCas9 cell lines, with clear enrichment of MitoCas9 in the mitochondrial fraction (M) compared to the cytoplasmic fraction (C). This is consistent with efficient expression of the MitoCas9, its translation in the cytoplasm, and subsequent import into mitochondria. As expected, the mitochondrial marker TOM20 was mainly found in the mitochondrial fraction and in total lysates.

Immunofluorescence staining was used to further verify mitochondrial localization of mitoCas9 (Fig. 2B). Cells were stained with anti-FLAG antibodies followed by Alexa Fluor 488 conjugated secondary antibodies. Although NARP3-1 mitoCas9 and NARP3-2 mitoCas9 cell lines express EGFP, the excitation and emission spectra of Alexa Fluor 488 and EGFP are highly similar. However, it is well known that fluorescent proteins exhibit significantly lower brightness and photostability than organic dyes. Moreover, aldehyde fixation of cells with 4% paraformaldehyde (PFA) can reduce GFP fluorescence due to partial protein denaturation^26,27^. Therefore, under conditions of relatively low EGFP expression, fixation with PFA, and the use of a bright dye such as Alexa Fluor 488, specific detection of target molecules is still feasible against background fluorescence. Negative staining controls of the NARP3-1 and NARP3-2 cybrid cell lines using an anti-FLAG antibody are presented in Supplementary Figure S4.

As a negative control, the parental NARP3-2 line was transiently transfected with the pEGFP-N1 plasmid, which lacks both a nuclear localization signal (NLS) and an MTS, resulting in diffuse cytoplasmic EGFP fluorescence. As a positive control for mitochondrial localization, the NARP3-2 cell line was transiently transfected with the pTurboGFP-mito plasmid, which encodes green fluorescent protein TurboGFP at the N-terminus fused to the COX8A MTS, similar to mitoCas9. To stain mitochondria, cells were co-transfected with the pTagRFP-mito plasmid, encoding a red fluorescent protein targeted to mitochondria.

Immunofluorescence analysis showed that most of NARP3-1 mitoCas9 and NARP3-2 mitoCas9 cells, the green fluorescence signal - corresponding to 3xFLAG-tagged Cas9 - that displayed a typical pattern of an intact mitochondrial network and colocalized with mitochondria (Fig. 2B). Confocal microscopy revealed overlapping green (3xFLAG-Cas9) and red (TagRFP-mito) fluorescence signals, consistent with mitochondrial import of mitoCas9. Given the inherent subjectivity of qualitative colocalization assessment, we performed quantitative colocalization analysis using ZEN 2010 software. Z-stack images were processed by maximum intensity projection, and overlapping regions in the red and green channels were visualized as yellow. The software automatically calculated the Pearson correlation coefficient (PCC) between red and green pixel intensities, applying the Costes threshold^24^ (Fig. 2B).

The PCC between the green (3xFLAG-Cas9) and red (TagRFP-mito) channels was 0.41 for the NARP3-1 mitoCas9 cell line and slightly higher at 0.50 for the NARP3-2 mitoCas9 line. These values were compared to the positive control (NARP3-2 cells co-transfected with pTurboGFP-mito and pTagRFP-mito), which had a PCC of 0.79, and the negative control (NARP3-2 cells co-transfected with pEGFP-N1 and pTagRFP-mito), which had a PCC of 0.23. The intermediate PCC values observed in both mitoCas9-expressing lines indicate spatial overlap between mitoCas9 and mitochondrial markers, supporting mitochondrial localization of the nuclease.

Although both cell lines express EGFP as part of the mitoCas9 construct integrated into their genomes (Fig. 1A), the PCC values for mitoCas9 (0.41 and 0.50) are approximately twice as high as that of the negative control (0.23). As noted previously, EGFP expression is higher in the NARP3-1 mitoCas9 line compared to NARP3-2 mitoCas9 (Supplementary Table 1), consistent with a lower PCC observed in the former. These findings further support that mitoCas9 is efficiently imported into the mitochondria of cybrid cells.

**Fig. 3:**
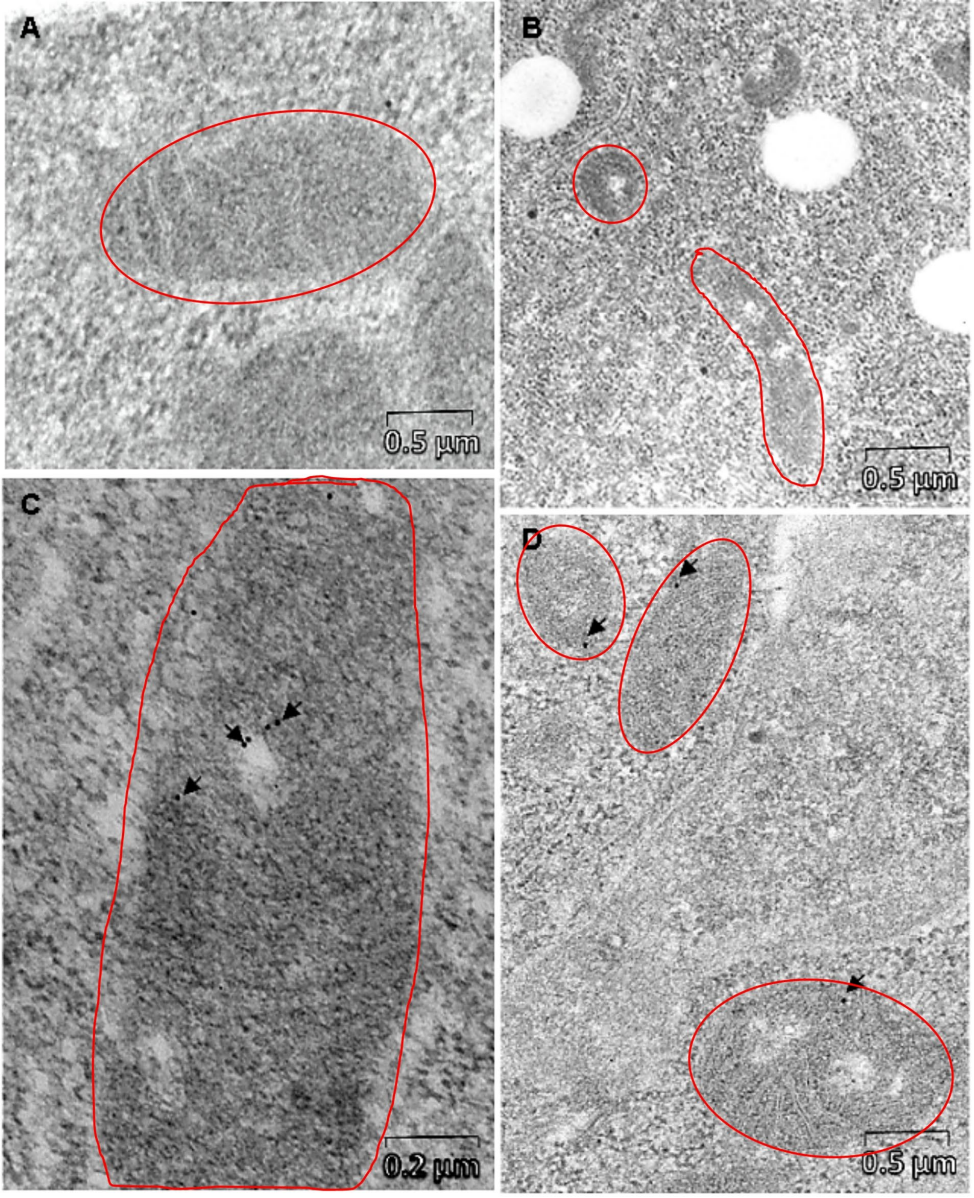
ImmunoEM images of mitochondria in cybrid cells NARP3-1 (**A**), NARP3-2 (**B**), NARP3-1 mitoCas9 (**C**) and NARP3-2 mitoCas9 (**D**). Mitochondria are outlined with a red line. Subcellular localization of mitoCas9 is indicated by the black arrow.

To perform its intended function - introducing double-strand breaks at target sites in mitochondrial DNA - Cas9 must localize to the mitochondrial matrix. However, western blot and immunofluorescence analyses alone cannot definitively resolve whether mitoCas9 localizes to the outer membrane, intermembrane space, inner membrane, or matrix. Therefore, we performed immunoelectron microscopy for higher-resolution localization. Gold-conjugated antibodies specifically detected mitoCas9 (via the 3xFLAG epitope) within the mitochondrial matrix of both NARP3-1 mitoCas9 and NARP3-2 mitoCas9 cell lines (Fig. 3).

In summary, we generated two cybrid cell lines, NARP3-1 mitoCas9 and NARP3-2 mitoCas9, harboring the m.8993T>G mutation at heteroplasmy levels of approximately 95% and 50%, respectively. These lines stably express mitoCas9, which is efficiently imported into the mitochondrial matrix.

### The effect of mitochondrial targeting of SpCas9 on mitochondrial health

**Fig. 4:**
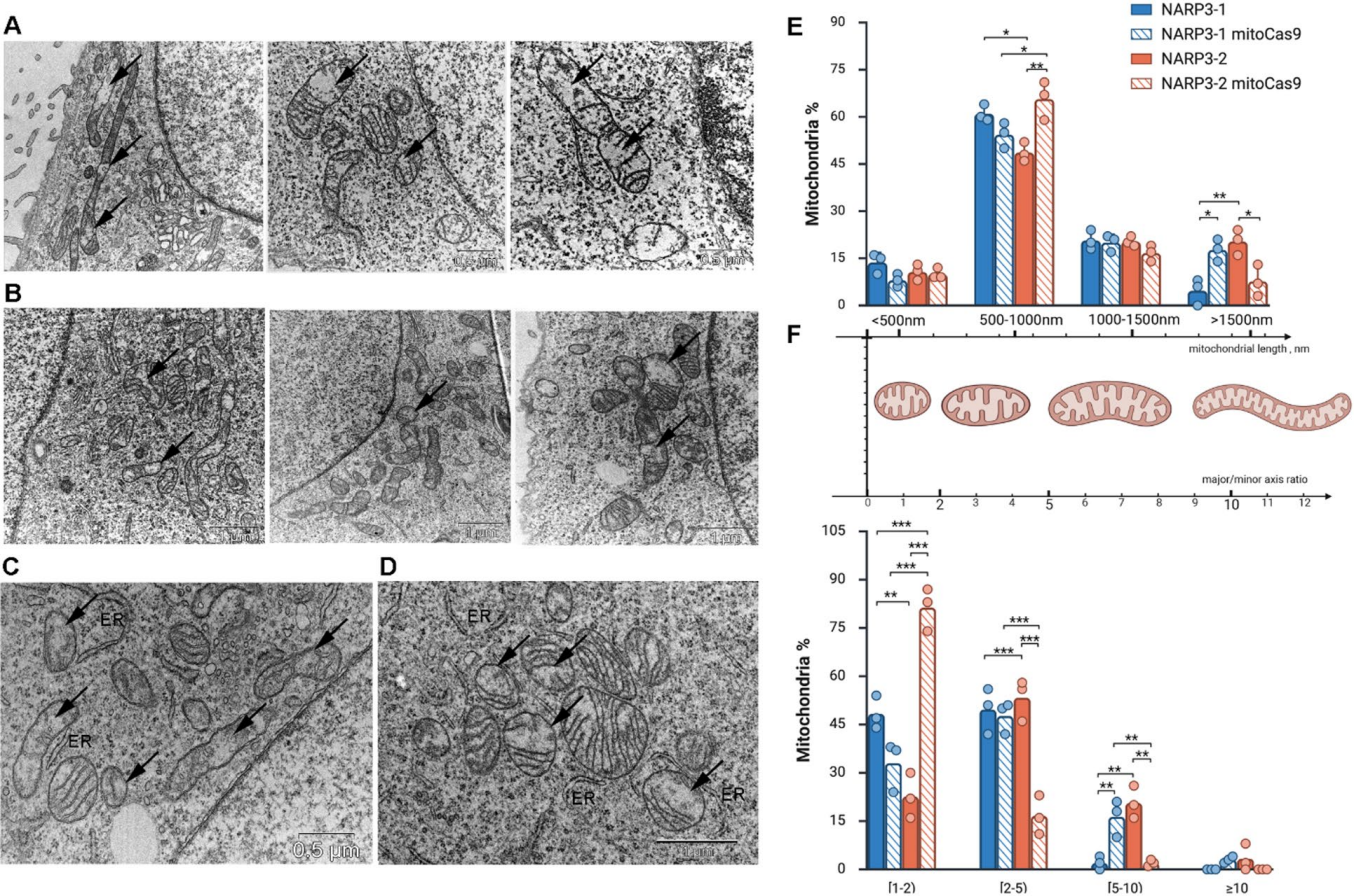
Effect of mitoCas9 on mitochondrial morphology. Ultrastructural electron microscopy analysis of cybrid cell lines: NARP3-1 (**A**), NARP3-2 (**B**), NARP3-1 mitoCas9 (**C**), NARP3-2 mitoCas9 (**D**). Arrows indicate light sparse areas of the mitochondrial matrix without cristae. ER - endoplasmic reticulum. Morphometric parameters of mitochondria in the parental and transgenic cybrid cell lines: mitochondrial length (**E**), circularity index (**F**). Values of circularity index: (1–2) – round mitochondria, (2–5) – oval mitochondria, (5–10) – elongated mitochondria, ≥ 10 – highly elongated mitochondria. *n* = 100, values for three independent biological replicates are presented as mean ± SD. Asterix depicts post hoc Tukey test p-value: * p-value < 0.05, **p-value < 0.01, *** p-value < 0.001; **** p-value < 0.0001. ns – non-significant ANOVA p-value.

Previous studies have demonstrated that the expression of nucleases^14^, as well as other proteins^28,29^ with mitochondrial localization, can cause mitochondrial damage. To evaluate the impact of mitoCas9 expression on mitochondrial integrity in the cybrid cell lines NARP3-1 mitoCas9 and NARP3-2 mitoCas9, we conducted ultrastructural analysis using transmission electron microscopy (Fig. 4). In both the parental and transgenic cybrid cell lines, varying degrees of matrix rarefaction were observed. The mitochondrial matrix in the NARP3-1 cell line appeared more rarefied compared to NARP3-2, which aligns with the higher heteroplasmy level of the m.8993T>G mutation. The cristae in mitochondria of the NARP3-2 cell line resembled typical parallel narrow structures more closely, whereas in NARP3-1 cells, abnormalities in cristae shape and organization were noted (partial absence of cristae and low density of the matrix of mitochondria), again correlating with the higher heteroplasmy level of the m.8993T>G mutation in NARP3-1 cells (Fig. 4A,B). Similar alterations in mitochondrial matrix were also observed in the transgenic cybrid cell lines expressing mitoCas9 (Fig. 4C,D).

Beyond matrix density and cristae structure, the morphology of the mitochondrial network also affects mitochondrial health^30^. Therefore, we analyzed mitochondrial length and circularity index in both parental and transgenic cybrid cell lines. For each parameter, normality was assessed by the Shapiro–Wilk test, which showed no significant deviation from normality in any group (*p* > 0.05), and the effects of NARP line and mitoCas9 expression were evaluated by two-way ANOVA. For mitochondrial length, the ANOVA revealed significant differences in the 500–1000 nm and ≥ 1500 nm groups (Fig. 4E).

Post hoc Tukey HSD analysis showed significant differences in the 500–1000 nm group between NARP3-1 and NARP3-2, between NARP3-2 and NARP3-2 mitoCas9, and between NARP3-1 mitoCas9 and NARP3-2 mitoCas9. In the ≥ 1500 nm group, significant differences were observed between the parental lines NARP3-1 and NARP3-2, as well as between parental and transgenic cybrid cell lines, specifically NARP3-1 vs. NARP3-1 mitoCas9 and NARP3-2 vs. NARP3-2 mitoCas9.

We also examined the circularity index, defined as the ratio of the minor to major mitochondrial axis. Values approaching 1 indicate spherical or rounded mitochondria, whereas higher values (up to ~ 10) indicate elongated morphology (Fig. 4F). Mitochondria with circularity values of 2–5 are considered morphologically oval, while values above 5, approaching 10, indicate long and highly elongated mitochondria.

For mitochondrial circularity, the Shapiro–Wilk test again showed no significant deviation from normality in any group (*p* > 0.05). The ANOVA revealed significant differences in the 1–2, 2–5, and 5–10 circularity groups (Fig. 4F). Post hoc analysis showed significant differences in the 1–2 and 5–10 groups between the parental lines NARP3-1 and NARP3-2 and between NARP3-2 and NARP3-2 mitoCas9, whereas the comparison between NARP3-1 and NARP3-1 mitoCas9 did not reach significance. In the 2–5 group, a significant difference was observed only between NARP3-2 and NARP3-2 mitoCas9, while NARP3-1 and NARP3-1 mitoCas9 did not differ significantly.

In summary, mitochondrial morphology analysis revealed significant differences both between NARP3-1 and NARP3-2 parental lines, likely due to their varying m.8993T>G heteroplasmy levels, and between parental and mitoCas9-expressing derivatives. Notably, the impact of mitoCas9 expression on mitochondrial length and shape was opposite in the NARP3-1 mitoCas9 and NARP3-2 mitoCas9 cybrid cell lines. In NARP3-1 mitoCas9, the number of long, elongated mitochondria increased, whereas in NARP3-2 mitoCas9, such mitochondria became less common, with a decrease in length and an increase in the number of rounder mitochondria.

### Targeting modified single guide RNA into mitochondria

The second component of the CRISPR-Cas9 system is the short single guide RNA (sgRNA), which determines the target sequence adjacent to where the Cas9 nuclease introduces a double-strand break. It is believed that certain RNA molecules within the cell can be imported into the mitochondrial matrix. This import process is aided by the presence of specific stem-loop structures within their sequence, such as the F and D domains of yeast tRNA^Lys(CUU)^ or the RP and MRP stem-loops found in the RNA components of corresponding ribonucleases. These domains can be used for RNA import into mammalian mitochondria^31^.

**Fig. 5:**
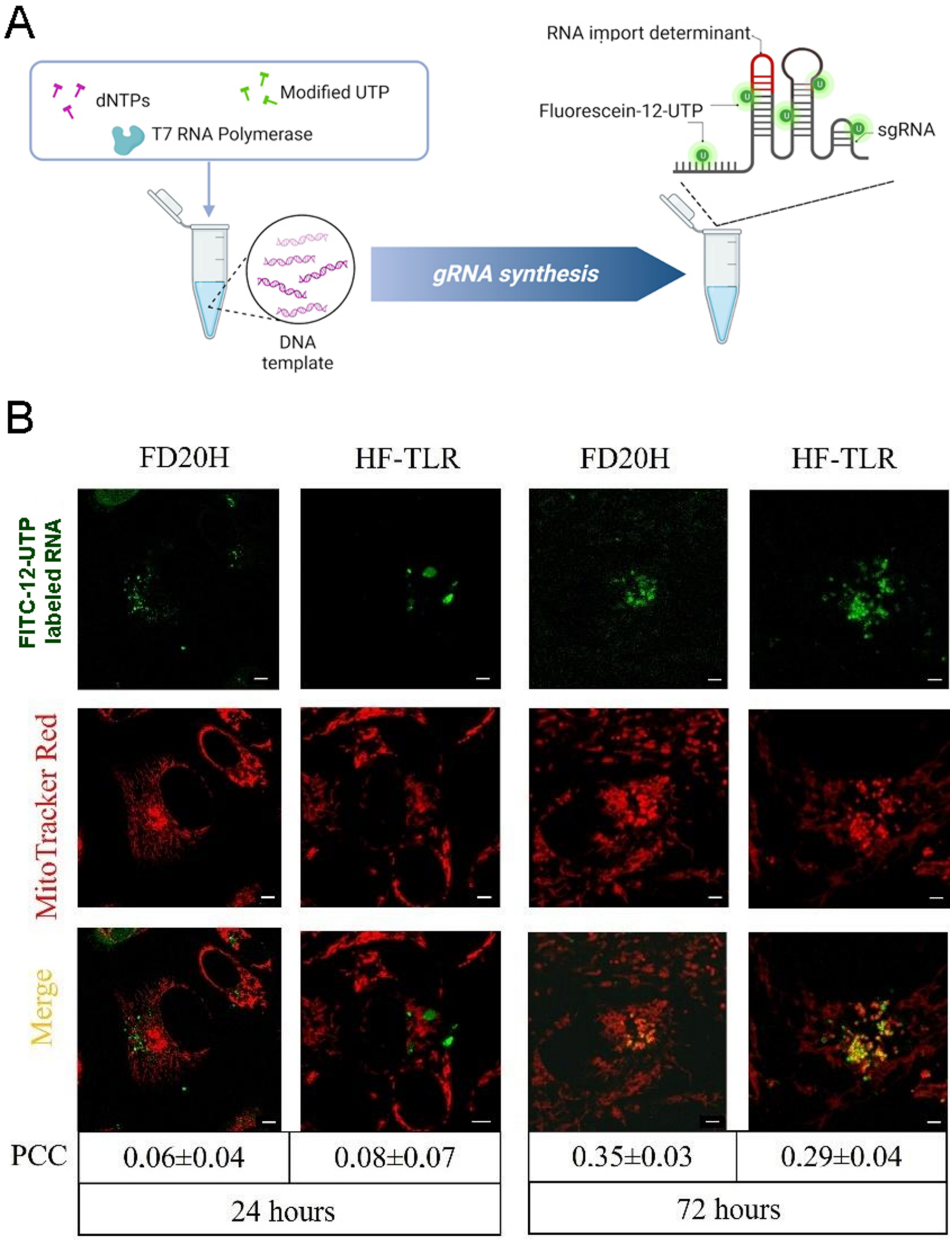
Import sgRNA into mitochondria. (**А**) A structure and synthesis of labeled sgRNA. (**B**) Subcellular localization of HF-TLR sgRNA and FD20H RNA using confocal laser scanning microscopy. HeLa cells were transiently transfected with FITC-12-UTP-labeled RNA and images were acquired at 24 and 72 h post-transfection. Mitochondria were stained with MitoTracker Red. PCC - Pearson correlation coefficient for green and red channel pixels. Scale bar, 5 μm.

Previously, we showed that inserting stem-loop structures F, D, RP, and MRP into the tetraloop or stem-loop 2 regions of sgRNA does not impair the functional activity of the CRISPR-Cas9 complex in vitro^19^. To assess whether such modified sgRNAs can be imported into the mitochondrial matrix, we used confocal microscopy. For this purpose, a fluorescently labelled sgRNA containing an F stem-loop in the tetraloop region (HF-TLR) was transiently transfected into HeLa cells. The colocalization of the labelled sgRNA HF-TLR with mitochondria stained with the fluorescent dye MitoTracker™ Red CMXRos was assessed by confocal microscopy at 24 and 72 h post-transfection (Fig. 5). As a positive control, we used labelled FD20H RNA from the study of Tonin Y et al.^32^. For quantitative analysis of the colocalization in the confocal microscopy images, we evaluated the Pearson correlation coefficient. At 24 h post-transfection, the signal from both sgRNA HF-TLR and FD20H RNA appeared as clusters of large granules in the cytoplasm, which did not colocalize with mitochondria (PCC = 0.06 and PCC = 0.08, respectively) (Fig. 5). However, at 72 h post-transfection, the signal from both sgRNA HF-TLR and FD20H RNA was partially detected within mitochondria, with PCC values of 0.29 and 0.35, respectively (Fig. 5). These results demonstrate that sgRNA HF-TLR is partially imported into the mitochondria.

### CRISPR-Cas9 system shifts mtDNA heteroplasmy in cybrid cells

For cleavage of mitochondrial DNA, both the modified sgRNA and mitoCas9 nuclease must be delivered to the mitochondrial matrix to form a functional complex. To evaluate CRISPR-Cas9-mediated cleavage of mtDNA, we used parental cybrid cell lines NARP3-1 and NARP3-2, as well as transgenic cybrid lines NARP3-1 mitoCas9 and NARP3-2 mitoCas9, which exhibit heteroplasmy for the m.8993T>G mutation at approximately 95% and 50%, respectively. The sgRNA was designed such that the m.8993T>G substitution is located two nucleotides downstream of the 3`-end of the protospacer, placing it at the second position of the NGG PAM motif required for mitoCas9 activity. Therefore, only the mutant mtDNA containing the m.8993T>G substitution has a functional PAM site and can be specifically cleaved, while the wild-type sequence remains unaffected.

**Fig. 6:**
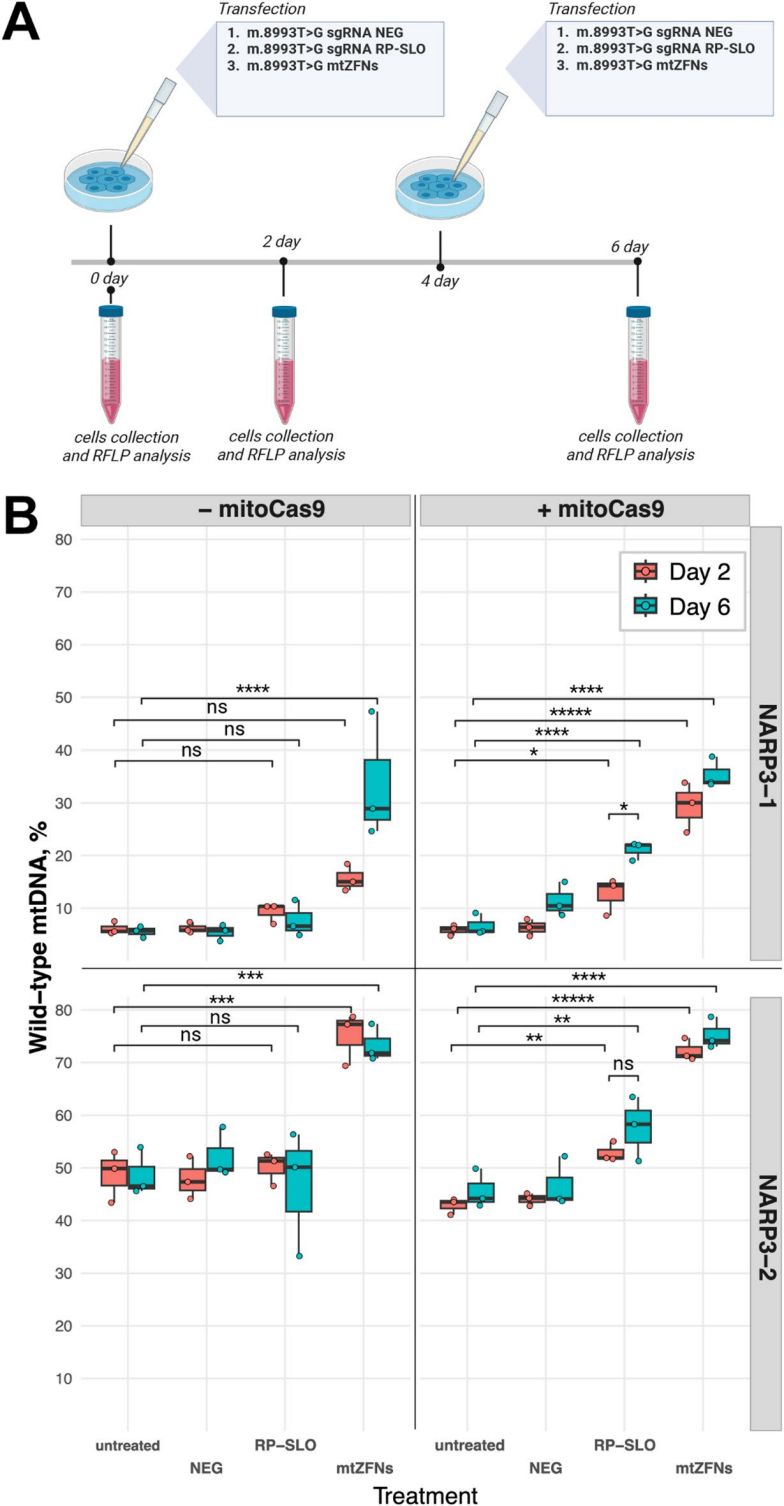
CRISPR-Cas9-Induced shift in mtDNA heteroplasmy. (**А**) A schematic overview of transfection and heteroplasmy assessment in cybrid cell lines. Transient transfections with sgRNAs and the ZFN system were performed on days 0 and 4 in three independent replicates. Heteroplasmy levels were analyzed on days 2 and 6 post-transfection by PCR-RFLP (**B**) Evaluation of mtDNA heteroplasmy level shifts by CRISPR-Cas9 and mtZFNs systems based on PCR-RFLP analysis in cybrid cell lines in vitro. Significant differences were evaluated using two-way analysis of variance (ANOVA) followed by post hoc pairwise comparisons, and are indicated by symbols above the plots. Data are presented as the mean ± SD; *n* = 9. Asterix depicts post hoc Tukey test p-value: * p-value < 0.05, **p-value < 0.01, *** p-value < 0.001; **** p-value < 0.0001. ns – p-value > 0.05. For original gel images, please refer to the supplementary materials (Fig. S5).

To express the sgRNA, cells were transiently transfected with a plasmid encoding the sgRNA RP-SLO, which includes the RP stem-loop in the forward orientation in stem loop 2^19^. As a negative control, we used a plasmid encoding an unmodified sgRNA (NEG) mitochondrial import determinants. Due to the relatively low efficiency of transient transfection in cybrid cells, transfection was performed twice, on days 0 and 4. The level of heteroplasmy was assessed by PCR-RFLP on days 2 and 6. The experimental design is illustrated in (Fig. 6A). In addition, untreated cells were used as negative controls, while cells transfected with plasmids encoding components of the mtZFN system, which specifically targets the m.8993T>G mutation, served as positive controls^9^.

By day 6, cells expressing RP-SLO sgRNA and mitoCas9 showed a significant shift in heteroplasmy toward wild-type mtDNA (Fig. 6B). In the NARP3-1 mitoCas9 cells transiently transfected with RP-SLO sgRNA, the proportion of wild-type mtDNA increased from approximately 5% initially to 12% by day 2 (*P* = 0.030) and reached 21% by day 6 (*P* = 5.3 × 10⁻⁵), corresponding to an overall shift of ~ 16% toward wild-type mtDNA. A similar pattern was observed in NARP3-2 mitoCas9 cells, where wild-type mtDNA levels increased from approximately 40% initially to about 53% by day 2 (*P* = 7.8 × 10⁻³), and to 60% by day 6 (*P* = 1.7 × 10⁻³), indicating a ~ 20% shift. In contrast, no significant changes in heteroplasmy were observed in untreated NARP3-1 or NARP3-1 mitoCas9 cells, nor in cells transfected with the negative control sgRNA NEG. As a positive control for heteroplasmy shift at the m.8993T>G site, we used previously validated mtZFNs specific to this mutation^9^. Treatment with mtZFNs resulted in a pronounced (~ 30%) shift in heteroplasmy toward wild-type mtDNA in all cybrid lines by day 6.

Taken together, these results clearly demonstrate that the CRISPR-Cas9 system specifically shifts m.8993T>G heteroplasmy toward wild-type mtDNA, albeit with lower efficiency compared to mtZFNs.

**Fig. 7:**
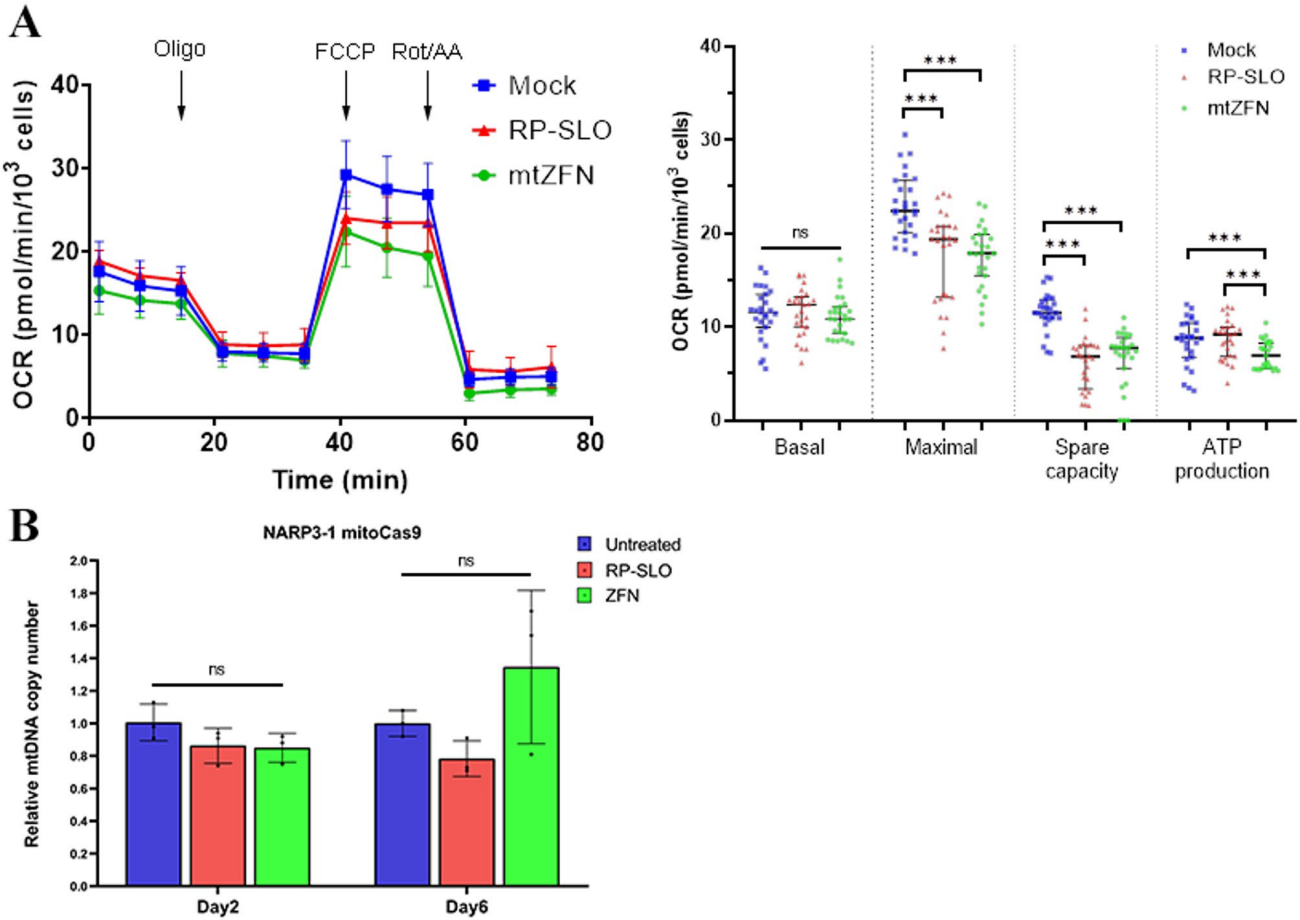
Mitochondrial respiration profiles and mtDNA copy number in NARP3-1 mitoCas9 cybrid cell line. (**A**) Respiration parameters were measured using the Seahorse XF Mito Stress Test (Agilent). Oligo – oligomycin; FCCP - carbonylcyanide-p-trifluoromethoxyphenylhydrazone; Rot/AA – rotenone/antimycin A. Transient transfections of NARP3-1 mitoCas9 cybrid cells with the sgRNA RP-SLO or mtZFN system were performed on days 0 and 4 and bioenergetic profiles were measured on days 6–8. Mock – transfection without nucleic acids. Seahorse OCR traces are shown to the left; mitochondrial respiration parameters are shown to the right. For OCR traces the values and error bars represent the mean ± SD of *n* = 3 independent biological replicates. For mitochondrial respiration parameters, the values and error bars represent the median with interquartile range of *n* = 3 independent biological replicates. Statistical significance was analyzed using Mann-Whitney test. (**B**) Mitochondrial DNA copy number in transgenic cybrid cell line NARP3-1 mitoCas9 transiently transfected with sgRNA RP-SLO or m.8993T>G-specific mtZFNs. Transient transfections with sgRNA and the ZFN system were performed on days 0 and 4 in three independent replicates. mtDNA copy numbers were quantified on days 2 and 6 post-transfection by real-time PCR. Data are presented as mean values ± SD (*n* = 3 technical replicates from 3 biological replicates). Statistical significance was analyzed using one-way ANOVA with post-hoc Tukey HSD test for multiple comparisons. *** *p*-value < 0.001; ns – not significant.

To assess mitochondrial function, the bioenergetic profile of the transgenic NARP3-1 mitoCas9 cell line stably expressing the mitoCas9 nuclease and transiently transfected with plasmids encoding either RP-SLO sgRNA or mtZFNs was evaluated using the Seahorse XF Cell Mito Stress Test (Fig. 7). We hypothesized that a shift in heteroplasmy induced by mitoCas9 or mtZFNs would lead to an altered mitochondrial respiration profile, measured as oxygen consumption rate (OCR). However, compared to mock-transfected controls, OCR profiles remained largely unchanged (Fig. 7A). Detailed analysis of mitochondrial respiration parameters revealed that, relative to mock controls, stable expression of mitoCas9 in combination with RP-SLO sgRNA caused a small but significant reduction in maximal respiration and spare respiratory capacity, while basal respiration remained unchanged (Fig. 7A). Similarly, expression of mtZFNs in NARP3-1 mitoCas9 cells led to comparable effects and a slight but significant decrease in ATP production (Fig. 7A).

Given that the initial heteroplasmy level in NARP3-1 mitoCas9 cells was approximately 95%, we hypothesized that the observed decrease in mitochondrial respiration parameters following CRISPR-Cas9 or mtZFN treatment might be due to mtDNA depletion. To test this hypothesis, we measured mtDNA copy number in the transgenic cybrid cell line NARP3-1 mitoCas9 after transient transfection with sgRNA RP-SLO and m.8993T>G-specific mtZFNs (Fig. 7B). The mtDNA copy number after CRISPR-Cas9 or mtZFN treatment did not differ significantly from that of the untreated control. However, following CRISPR-Cas9 treatment on days 2 and 6, and mtZFN treatment on day 2, a trend toward an approximately 15% reduction in mtDNA copy number was observed, which may indicate moderate specific activity. It should be noted that in the original study^9^, m.8993T>G-specific mtZFNs likewise did not lead to a reduction in mtDNA copy number. Only after FACS-based selection of cybrid cells exhibiting high levels mtZFN expression was a shift in heteroplasmy toward wild-type mtDNA achieved, which was also accompanied by mtDNA depletion^8^. Taken together, the analysis of mitochondrial respiration parameters and mtDNA copy number following CRISPR-Cas9 or mtZFN treatment indicates that, although a 16–30% shift toward wild-type mtDNA can be achieved, the activity of both systems remains moderate and may not be sufficient to restore mitochondrial respiratory function under these conditions.

## Discussion

In this study, we demonstrate the functional activity of a mitochondria-targeted CRISPR-SpCas9 system engineered to selectively shift heteroplasmy level at the m.8993T>G mutation in human cybrid cells. We confirmed mitochondrial localization of the SpCas9 nuclease and effective delivery of modified single guide RNAs containing import determinants. The application of the system resulted in a consistent shift in mtDNA heteroplasmy toward the wild-type allele by approximately 16–20% over six days. Despite this molecular correction, no significant improvement in mitochondrial respiratory function or mtDNA depletion was observed within the same timeframe. These findings establish the feasibility of using CRISPR-SpCas9 for allele-specific manipulation of pathogenic mtDNA variants, while highlighting current limitations in functional rescue.

To our knowledge, this is the first study to achieve a specific and reproducible shift at the m.8993T>G locus using а mitochondria-targeted CRISPR-SpCas9 system. While previous genome-editing strategies, including mtZFNs, mitoTALENs, and mitochondria-targeted restriction endonucleases, have shown efficacy in eliminating mutant mtDNA, they require labor-intensive protein engineering and are limited in their programmability^4^. In contrast, our approach leverages the modularity and sequence flexibility of the CRISPR-Cas9 platform, relying on a single SpCas9 nuclease combined with easily customizable single guide RNAs engineered for mitochondrial import. This design facilitates rapid retargeting to different pathogenic mutations and enables broader application without the need to re-engineer the nuclease itself. Our findings thus expand the toolkit for precision mitochondrial genome manipulation and lay the foundation for further optimization of RNA-guided approaches.

Compared to previous strategies aimed at correcting this mutation, our approach introduced a distinct technological advance with a different trade-off profile. Initial studies employed mitochondria-targeted restriction enzymes such as SmaI and XmaI, which showed proof-of-concept efficacy but were limited by their dependence on unique restriction sites^6,7^. Subsequent work with mitochondrially targeted zinc-finger nucleases (mtZFNs), notably by the Minczuk laboratory, achieved a more pronounced correction, reducing mutant heteroplasmy from ~ 80% to ~ 7%, substantially surpassing the absolute shift obtained in our system^8^. The superior efficiency of mtZFNs likely stems from more robust mitochondrial import of the nuclease, higher target-site binding specificity, and independence from strict sequence constraints. In contrast, SpCas9 requires the presence of NGG protospacer adjacent motifs (PAMs), which limits the number of accessible sites in the compact mitochondrial genome and can constrain guide RNA design^33^. Despite these limitations, the CRISPR-SpCas9 platform offers important advantages, including its modularity, ease of reprogramming to different mutations via guide RNA redesign, and the extensive characterization of SpCas9 as a genome-editing tool. Importantly, the heteroplasmy shift we observed in our study was specific, reproducible, and statistically significant.

The feasibility of CRISPR-Cas-based editing of the mitochondrial genome has been widely debated, primarily due to uncertainties regarding the import of RNA into mitochondria^34^. Meanwhile, several recent studies have explored different Cas9 variants and RNA configurations. For example, Bi et al.^35^, used a similar mito-SpCas9 construct but unmodified guide RNAs to mediate low-efficiency knock-in (0.03–0.23%) of exogenous DNA in human cells^35^. Hussain^36^, employed a dual-targeting MLS1/MLS2 Cas9 and RP-loop-modified guide RNAs, reporting decreased mtDNA levels and transcript suppression, though without directly measuring heteroplasmy^36^. Schmiderer^37^, tested multiple RP-loop sgRNAs with mitochondrially localized SpCas9 but observed no editing or depletion, suggesting that guide RNA delivery, rather than Cas9 activity, remains the principal bottleneck^37^. Notably, the differences in editing efficiency between our study and those by Schmiderer et al., and Hussain et al., may also stem from the design strategy: while both groups appended RP-loop motifs to the 5′ or 3′ ends of the single guide RNA, we first modeled the secondary structure of our sgRNA in silico and incorporated the import determinant (RP-SLO) directly into the stem-loop region of the sgRNA scaffold. This internal placement preserves structural integrity and likely supports more effective mitochondrial import without compromising Cas9 complex activity, as previously demonstrated in vitro^19,16^. reported mtDNA depletion only when dual sgRNAs were used with structured motifs^38^. Wang^39^, achieved site-specific InDel formation in mtDNA using SaCas9 with a mitochondrial targeting sequence, but did not assess effects on heteroplasmy or function^39^. A summary of the published CRISPR-based mitochondrial editing systems, including nuclease and guide RNA designs, validation methods, and observed outcomes, is provided in Supplementary Table 3.

Although our guide RNAs were engineered with mitochondrial import determinants, we did not directly quantify mitochondrial uptake of the RP-SLO guide RNA used in functional assays. Instead, mitochondrial import was demonstrated using a related construct, HF-TLR, which has shown robust mitochondrial localization. The decision to use HF-TLR for import validation and RP-SLO for functional studies was based on prior evidence that both designs, differing only in the nature and position of their import motifs, support comparable SpCas9 activity^19^. Since the primary aim of this study was to evaluate shift of heteroplasmy, RP-SLO was selected for downstream application due to its superior performance in cleavage assays. While the observed heteroplasmy shift provides indirect support for mitochondrial delivery of RP-SLO, future work should include direct visualization or biochemical confirmation of its import.

Interestingly, our recent studies using CRISPR-Cas12a system further support the idea that guide RNA can be delivered into mitochondria without the need for extensive engineering. In the work by Nikitchina et al.^40^, mitochondrially targeted AsCas12a, guided by short unmodified crRNAs (~ 40 nt), successfully induced site-specific deletions in human mtDNA^40^. Previously we demonstrated that these crRNAs could be imported into the mitochondrial matrix without additional import signals^20^. This contrasts with our current findings using SpCas9, where unmodified single guide RNAs failed to elicit a heteroplasmy shift, and only RP-SLO-modified guides proved effective. These differences likely reflect intrinsic properties of the systems: Cas12a relies on compact crRNAs, which may bypass mitochondrial import barriers more efficiently than the ~ 100 nt sgRNAs used by SpCas9. Thus, while Cas12a-based systems may operate with unmodified RNA guides, our results suggest that SpCas9 systems still benefit from, or require, engineering of guide RNAs to ensure mitochondrial delivery and functionality.

The CRISPR/Cas system is a highly effective and widely used tool for nuclear genome editing. The introduction of a double-strand break (DSB) at a target genomic locus by this system activates endogenous cellular repair mechanisms. The two principal DSB repair pathways are the error-free, template-dependent homology-directed repair (HDR) pathway and the error-prone, template-independent non-homologous end joining (NHEJ) pathway. By exploiting these endogenous repair processes, CRISPR/Cas enables precise sequence modification, correction or modeling of disease-associated mutations, as well as gene knockout through the generation of insertions and deletions (indels), or larger genomic rearrangements^41^.

In contrast, the mechanisms of mtDNA repair are far less well understood than those operating in the nuclear genome, and it is widely accepted that mitochondria possess a more limited repertoire of DNA repair pathways than the nucleus. Only a subset of proteins involved in mammalian DSB repair has been shown to localize to mitochondria. Consequently, NHEJ is generally considered non-functional in this organelle, whereas DSB repair may occur through alternative mechanisms such as microhomology-mediated end joining or, potentially, HDR^42^. However, the existence and functional significance of these repair pathways in mitochondria remain unclear, and available evidence suggests that, even if present in mammalian mitochondria, they operate far less efficiently than DNA repair mechanisms in the nucleus. It is also important to consider that following DSB formation, linearized mitochondrial DNA is rapidly degraded by components of the mitochondrial replication machinery^21^.

Although several studies have reported low-frequency indel formation or knock-in of exogenous DNA into mtDNA^35,39,43^, the molecular mechanisms underlying these processes remain poorly understood. Collectively, these features of mitochondrial DNA repair biology explain why CRISPR/Cas systems are currently more suitable for shifting heteroplasmy through selective elimination of mutant genomes rather than for precise correction or introduction of mutations in mtDNA.

Mitochondrial DNA base editing has recently emerged as a powerful alternative to nuclease-based approaches for targeted manipulation of mtDNA, particularly for correcting pathogenic point mutations without inducing double-strand breaks. Similar to mitoZFNs and mito-TALENs, mitochondrial base editors consist of a DNA-targeting domain, such as a zinc-finger or transcription activator-like effector domain, fused to a deaminase enzyme that catalyzes cytidine or adenine deamination, thereby enabling direct C•G-to-T•A or A•T-to-G•C conversions in mtDNA^44–46^. Consequently, mitochondrial base editors provide a clear advantage for introducing or correcting specific point mutations without triggering mtDNA degradation.

Mitochondrial base editors have been successfully applied to correct pathogenic mtDNA point mutations associated with mitochondrial diseases and to generate animal models^47–49^. However, despite their advantages, mitochondrial base editors have several limitations, including off-target deamination in both mitochondrial and nuclear genomes, bystander edits within the editing window, sequence preference constraints, variable editing efficiency, a limited range of possible base substitutions, and size-related delivery challenges. The incorporation of additional protein domains, such as nickases or uracil glycosylase inhibitors, as well as advances in de novo protein design, has substantially improved the performance of mitochondrial base editors^48,50^. Importantly, unlike nuclease-based approaches, mitochondrial base editors can be used to edit homoplasmic mutations without inducing mtDNA depletion or cellular toxicity. Therefore, considering the respective strengths and limitations of mitochondrial base editing and nuclease-based strategies, the CRISPR-SpCas9 platform described here is primarily suited for heteroplasmy shifting through selective elimination of m.8993T>G mtDNA and offers greater programmability at the RNA-guided targeting level. Accordingly, base editing and nuclease-based approaches represent complementary strategies, each providing distinct mechanistic advantages and being optimal for different applications in mitochondrial genome engineering.

An important feature of the present study is the use of cybrid cell lines with stable expression of mitochondrially targeted SpCas9. This experimental strategy ensured consistent nuclease availability within the mitochondrial matrix and minimized variability associated with transient transfection of the mitoCas9 vector in cybrid cells. As demonstrated by PCR-RFLP analysis across 25 passages, stable mitoCas9 expression alone did not alter m.8993T>G heteroplasmy levels, indicating that nuclease presence in mitochondria is not sufficient to induce mtDNA cleavage in the absence of a specific sgRNA.

The ultrastructural and morphometric analyses revealed that mitoCas9 expression was associated with measurable changes in mitochondrial morphology, although these effects differed between NARP3-1 and NARP3-2 backgrounds. Importantly, these alterations did not translate into spontaneous changes in mtDNA heteroplasmy during long-term cultivation, suggesting that mitoCas9 expression does not cause progressive mtDNA instability.

The use of cybrid cell lines with a stable mitoCas9 background likely facilitated detection of the heteroplasmy shift upon transient delivery of RP-SLO sgRNA by ensuring continuous nuclease availability for ribonucleoprotein complex formation. However, despite this design, the magnitude of heteroplasmy shift achieved with CRISPR-Cas9 remained moderate (approximately 16–20%) and was lower than that observed with mtZFNs (~ 30%). This suggests that, while stable mitoCas9 expression provides a robust proof-of-principle platform, further optimization of sgRNA delivery, mitochondrial import efficiency, and cleavage activity will be required to enhance editing efficiency.

In our study, despite a significant molecular shift in heteroplasmy toward wild-type mtDNA, this molecular correction did not result in measurable improvement of mitochondrial respiratory function within the six-day experimental timeframe. Several biological factors may account for this discrepancy. First, the degree of heteroplasmy correction (16–20%) may fall below the phenotypic threshold required to restore oxidative phosphorylation, particularly for mutations such as m.8993T>G, where functional rescue typically requires a mutant load below 60%^51^. Second, the relatively short duration of post-editing observation may be insufficient for functional recovery, given the slow turnover and replication dynamics of mitochondrial genomes and respiratory complexes. To improve the therapeutic potential of the CRISPR-SpCas9 system, future studies should extend the duration of post-editing culture, explore repeated single guide RNA delivery, and enhance the efficiency of mitochondrial import using optimized RNA structures or protein chaperone systems. These efforts will help determine whether more robust shifts in heteroplasmy can lead to durable functional rescue.

While our results support the specificity and feasibility of CRISPR-SpCas9-mediated editing, the safety profile of this system remains to be fully characterized. In the present study, we did not perform a comprehensive assessment of off-target cleavage events within the mitochondrial genome. Given the compact nature of mtDNA and the limited availability of protospacer adjacent motifs (PAMs), unintended editing at homologous or partially matching sites could potentially disrupt essential genes or regulatory regions. Future studies employing high-depth mtDNA sequencing or sensitive off-target detection assays will be critical to ensure genomic integrity and therapeutic safety. Additionally, although we did not observe overt cytotoxicity during the experimental window, the sustained expression of mitochondria-targeted SpCas9 and repeated guide RNA delivery may elicit mitochondrial stress over longer periods. Evaluating cell viability, mitochondrial dynamics, and transcriptional responses over extended time courses will be essential to establish the long-term tolerability of the system. Addressing these safety concerns is a necessary step toward the potential clinical translation of CRISPR-based mitochondrial therapies.

## Conclusions

In this study, we demonstrate for the first time that a mitochondria-targeted CRISPR-SpCas9 system can achieve a specific and reproducible shift in mitochondrial DNA heteroplasmy at the pathogenic m.8993T>G mutation site in human cybrid cells. By combining a mitochondrially localized SpCas9 nuclease with engineered single guide RNA carrying mitochondrial import signal, we achieved a ~ 16–20% shift toward wild-type mtDNA within six days. Despite the successful heteroplasmy shift, it did not lead to a measurable improvement in mitochondrial respiratory function over the short experimental timeframe. These findings highlight both the promise and current limitations of CRISPR-based mitochondrial genome editing: while the system is programmable, modular, and mutation-specific, further optimization is needed to enhance efficiency, ensure functional rescue, and evaluate long-term safety. Our results expand the mitochondrial gene-editing toolkit and establish a foundation for developing RNA-guided approaches to target pathogenic mtDNA mutations. Future work should focus on improving guide RNA import, prolonging post-editing recovery, and rigorously assessing off-target effects to move closer to clinical applications.

## Supplementary Information

Below is the link to the electronic supplementary material.

Supplementary Material 1

Supplementary Material 2

## Data Availability

All data needed to evaluate the conclusions of this study are included in the main text and the Supplementary Information. Source data underlying the graphs and charts presented in this study are provided with this paper. Additional datasets used and/or analysed during the current studyare available from the corresponding author upon reasonable request.

## Abbreviations

mitoZFs: Mitochondrial zinc-finger nucleases
mito-TALENs: Mitochondrial transcription activator-like effector nucleases
mtDNA: Mitochondrial DNA
MTS: Mitochondrial targeting sequence
NARP: Neuropathy, Ataxia, and Retinitis Pigmentosa syndrome
NLS: Nuclear localization signal
PAM: Protospacer adjacent motif
PBS: Phosphate-buffered saline
PCC: Pearson correlation coefficient
PFA: Paraformaldehyde
RT: Room temperature
sgRNA: Single guide RNA
TEM: Transmission electron microscopy
