# Supplementary material for: Specific elimination of m.8993T>G mitochondrial haplotype in NARP cybrid cells by CRISPR-Cas9 system
Source: Sci Rep. 2026 Apr 28;16:19745. doi: 10.1038/s41598-026-49007-y (PMC13316044; doi:10.1038/s41598-026-49007-y)

**Supplementary Figure S1: Original full-length gel images used in Figure 1B for the evaluation of mtDNA heteroplasmy level with irrelevant lanes crossed out.** PCR-RFLP analysis of m.8993T>G heteroplasmy levels in parental and the transgenic clones NARP3-1 mitoCas9 and NARP3-2 mitoCas9. Total DNA isolated from cells was amplified by PCR. The presence of 960 bp and 718 bp bands indicates the presence of the copies of mtDNA carrying the m.8993T>G mutation site recognised by the AvaI restriction enzyme. NARP3-1 or NARP3-2 – parental cydrid cell lines. NARP3-1 mitoCas9 and NARP3-2 mitoCas9 – transgenic clones that stably express mitoCas9 nuclease. DNA isolated from the 2SA cell line, which does not contain the m.8993T>G mutation, was used as a negative control. M represents a 1kb Ladder (SibEnzyme).

1. NARP3-1/NARP3-1 mitoCas9


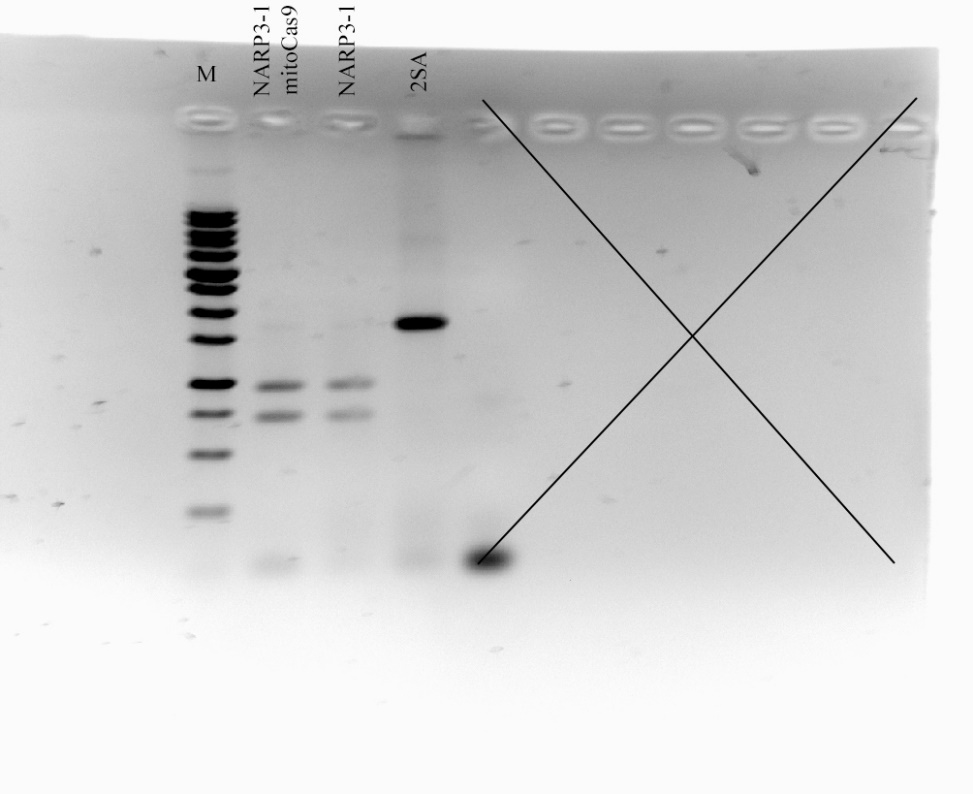


1. NARP3-2/NARP3-2 mitoCas9


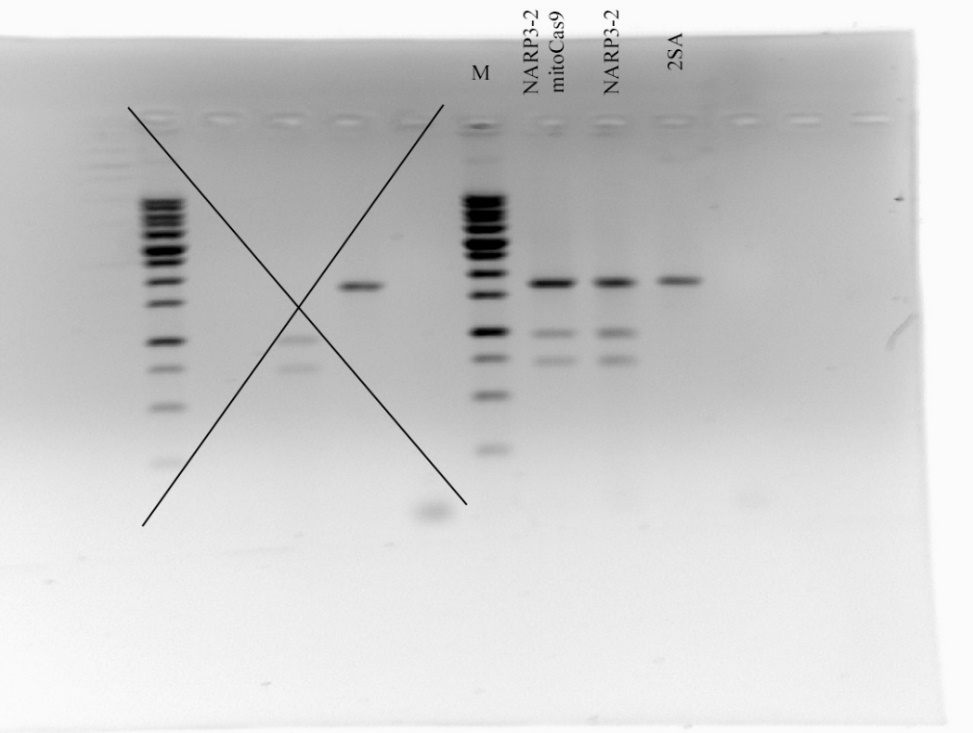


**Supplementary Figure S2: Original full-length gel images used in Figure 1C for the evaluation of mtDNA heteroplasmy level.** PCR-RFLP analysis of mtDNA heteroplasmy dynamics in parental and transgenic NARP cell lines across passages p5, p15 and p25. Total DNA isolated from cells was amplified by PCR. The presence of 960 bp and 345 bp bands indicates the presence of the copies of mtDNA carrying the m.8993T>G mutation site recognised by the AvaI restriction enzyme. DNA isolated from the 2SA cell line, which does not carry the m.8993T>G mutation, was used as a negative control. M represents a 100 bp Plus Ladder (SibEnzyme). Data are presented as three biological replicates (n = 3), each analyzed in three technical triplicates (given in brackets).

1. NARP3-1

**
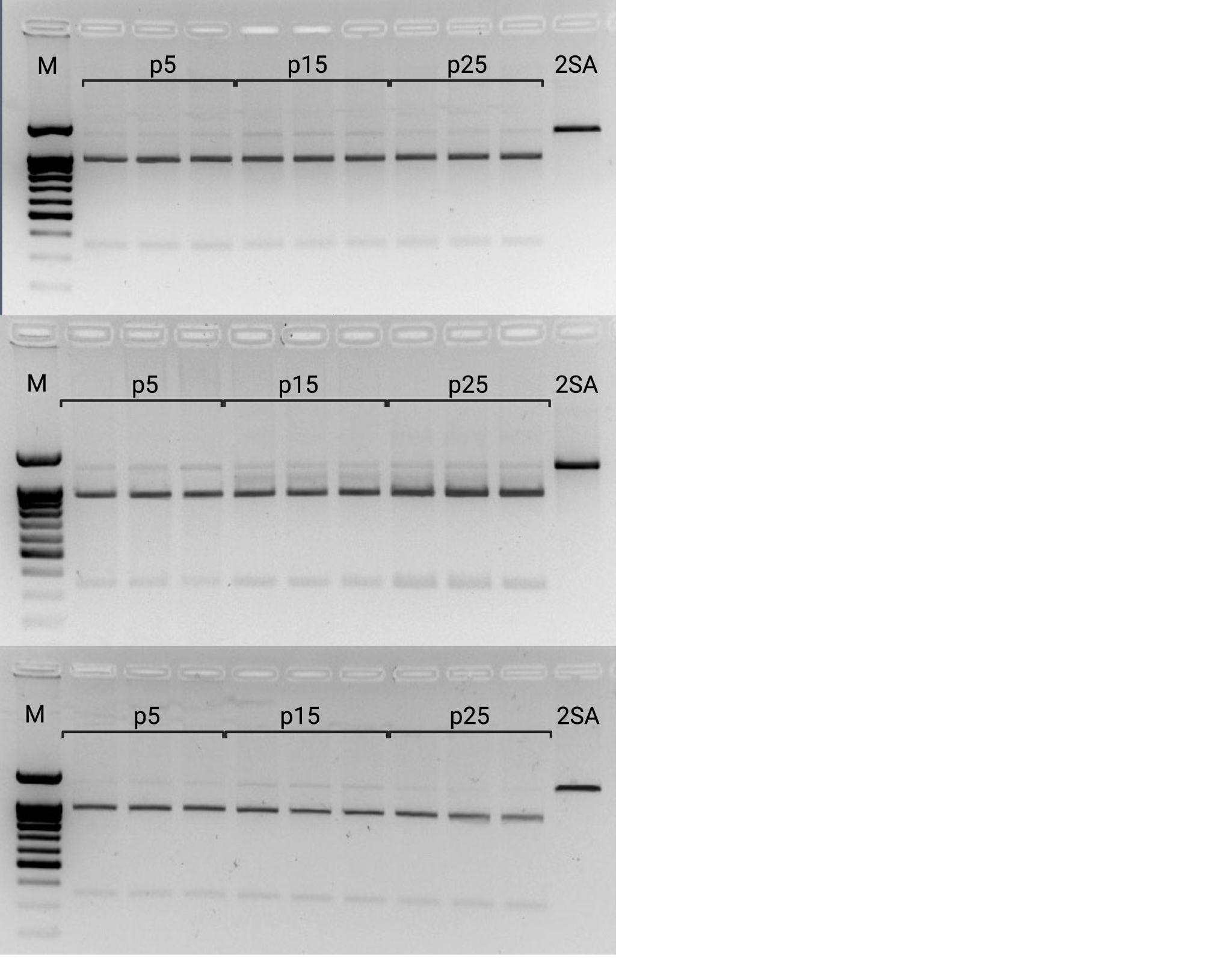
**

1. NARP3-1 mitoCas9

**
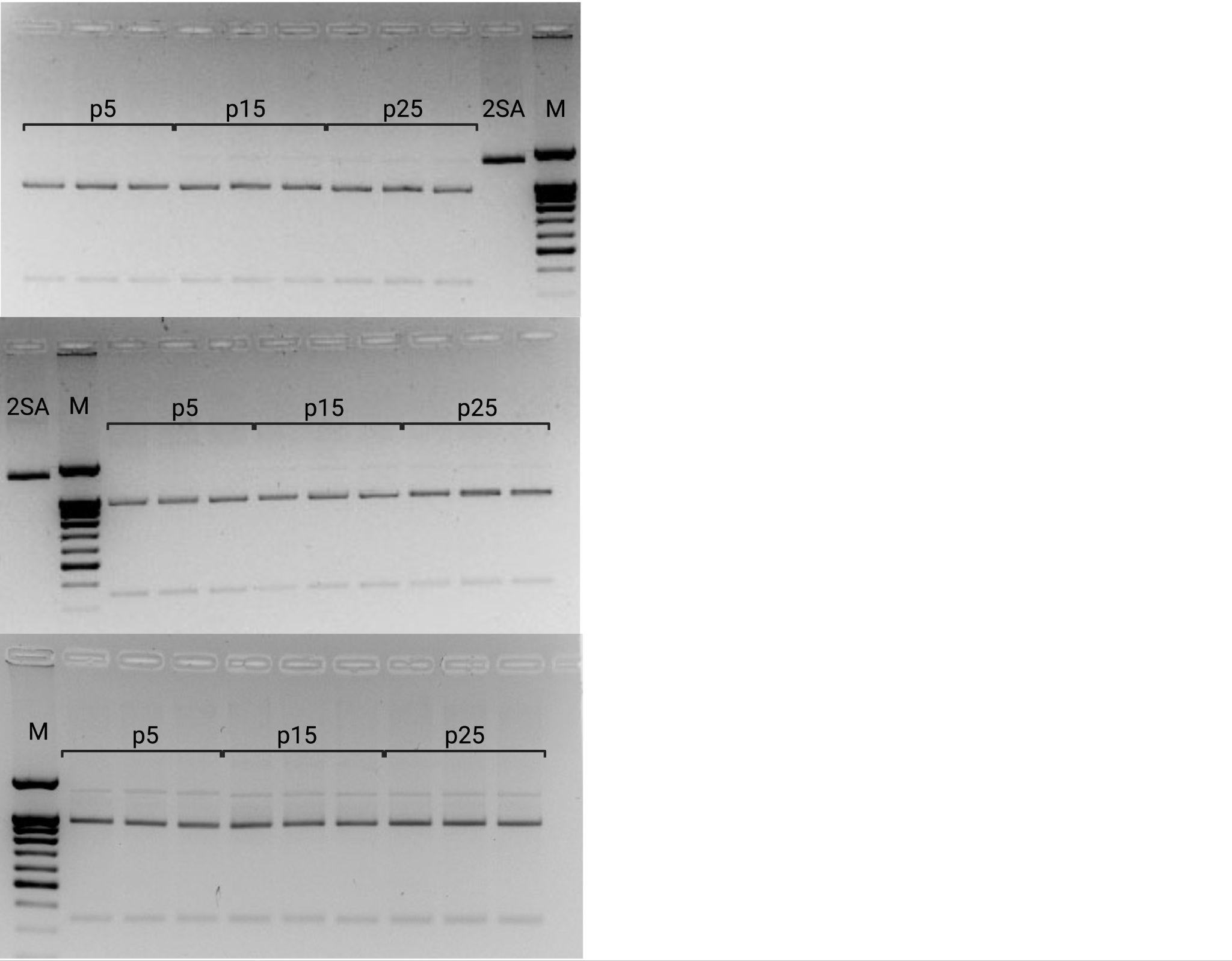
**

1. NARP3-2

**
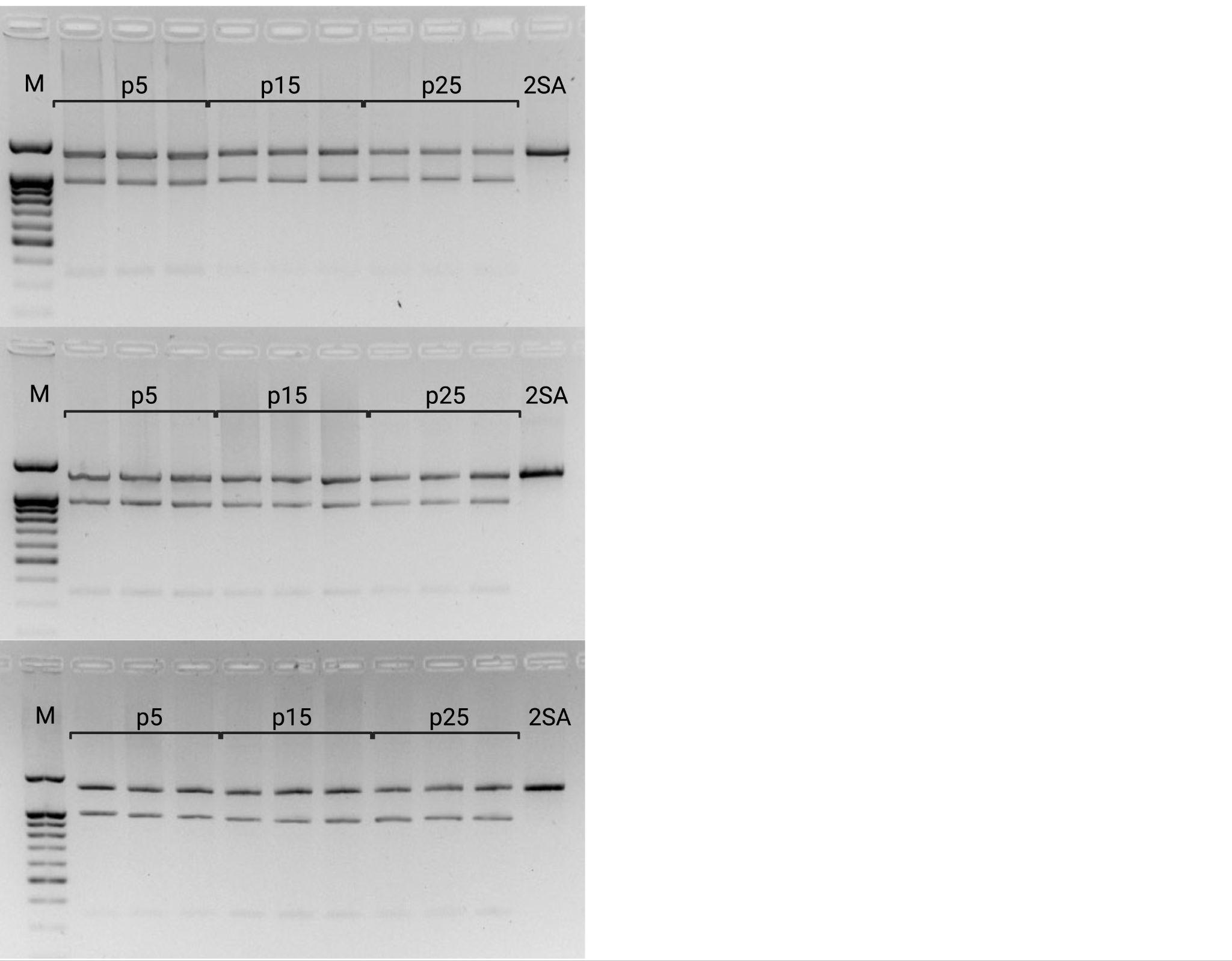
**

1. NARP3-2 mitoCas9

**
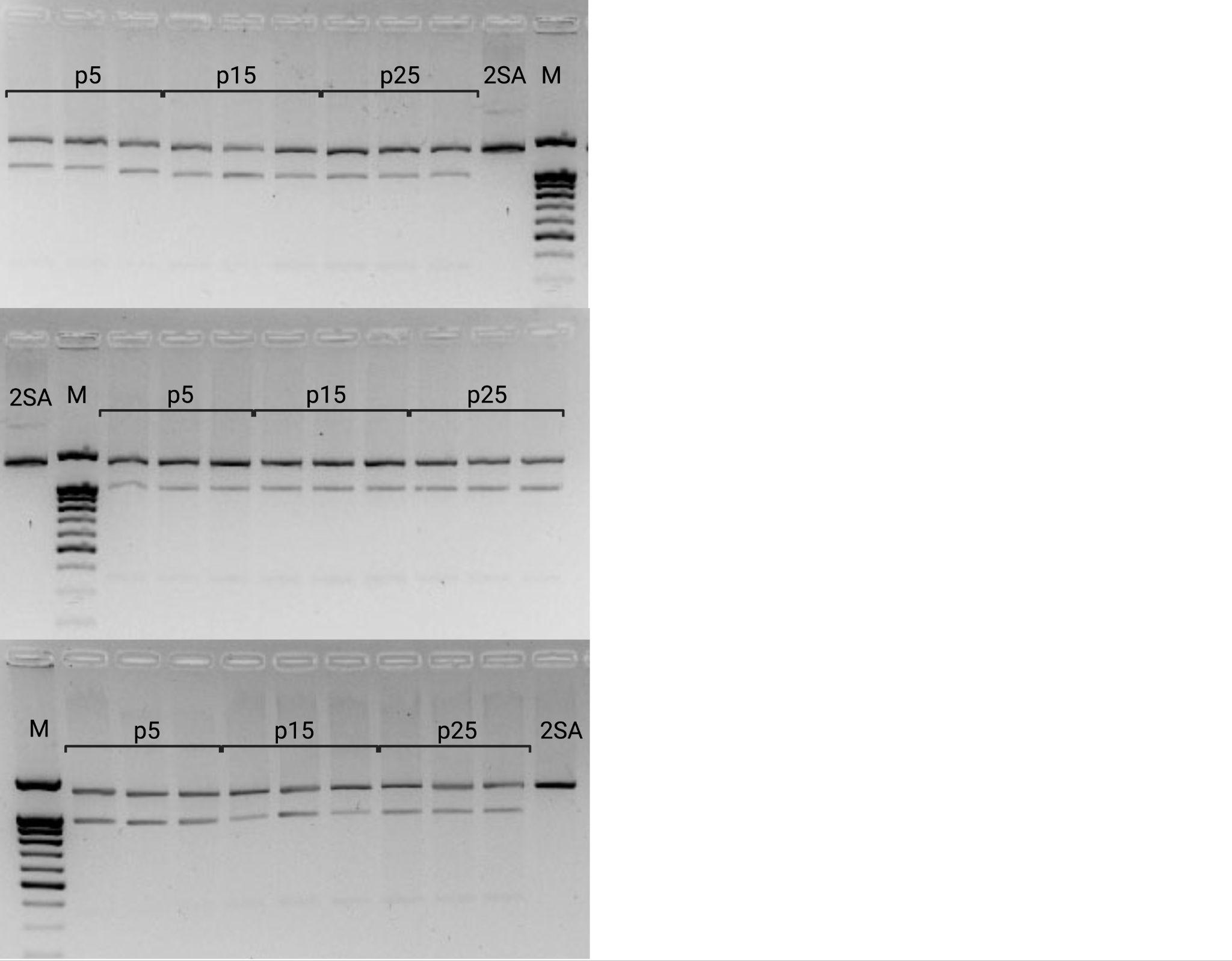
**

**Original images of Western-blot analyses:** To determine presence of multiple antigens in parallel, the protein-bound membranes were stripped twice for 10 min in Stripping buffer (200 mM glycine, 0.1% SDS, 1% Tween20, pH 2.2) at room temperature to remove bound antibodies. After washing twice for 10 min with PBS and twice for 5 min with TBST, the membranes were exposed to another set of antibodies.

Chemiluminescence signals were always directly scanned using the iBright™ FL1500 Imaging System (Invitrogen™), a black-and-white image of the membrane containing the prestained size marker proteins (iBright™ Prestained Protein Ladder (#LC5615, Invitrogen™) and PageRuler™ Prestained Protein Ladder (#26616, Invitrogen™) was taken in parallel. Prestained protein ladder mobility was calibrated against the unstained ladder PageRuler™ Unstained Protein Ladder (#26614, Thermo Scientific™).

**Supplementary Figure S3: Original images of Figure 2A**

**1)** Protein expression of **3xFLAG-Cas9** in parental **NARP3-1** and transgenic **NARP3-1 mitoCas9** cell lines determined by Western blotting. Left image - chemiluminescence signals, right image – overlay of chemiluminescence signals with the black-and-white image of the membrane. T – total cell lysate, C – cytoplasmic protein fraction, M – mitochondrial protein fraction. The arrow indicates the specific FLAG-Cas9 band. M1 – iBright™ Prestained Protein Ladder (#LC5615, Invitrogen™). M2 – PageRuler™ Prestained Protein Ladder (#26616, Invitrogen™). Saturated pixels are marked in red.


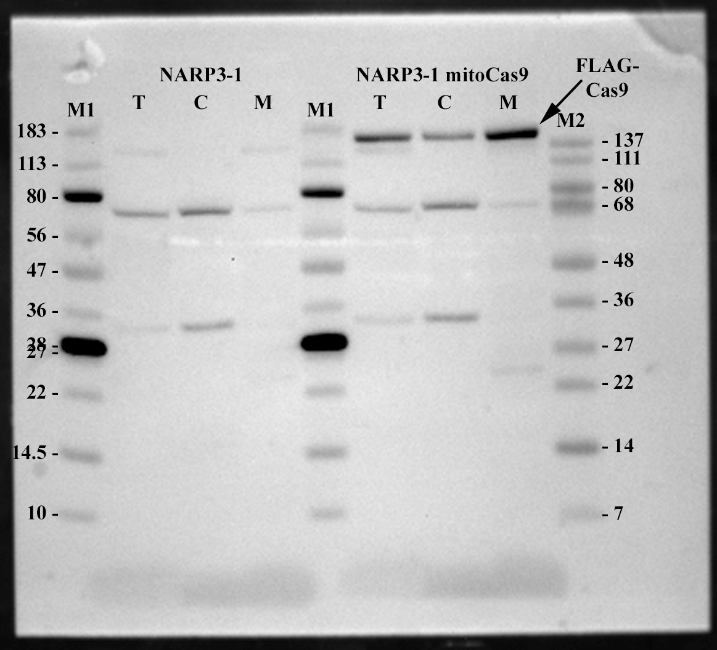

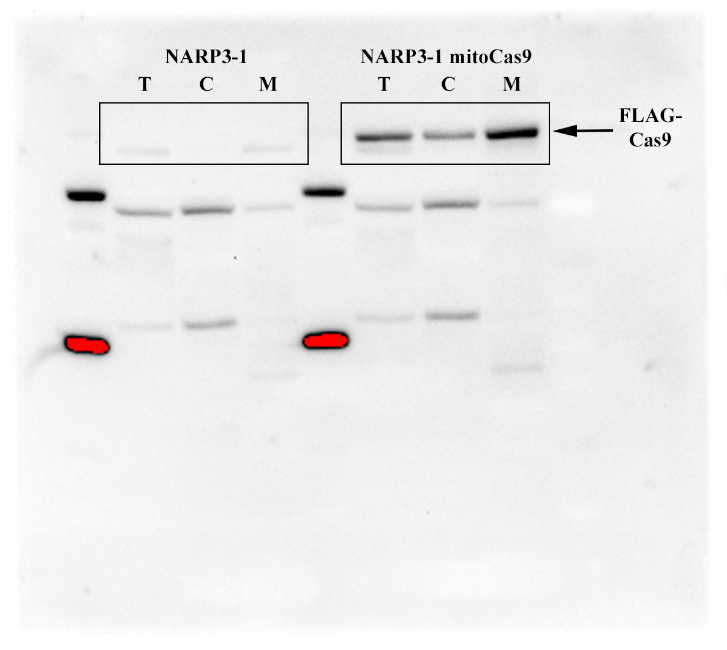


**2)** Protein expression of **3xFLAG-Cas9** in parental **NARP3-2** and transgenic **NARP3-2 mitoCas9** cell lines determined by Western blotting. Left image - chemiluminescence signals, right image – overlay of chemiluminescence signals with the black-and-white image of the membrane. T – total cell lysate, C – cytoplasmic protein fraction, M – mitochondrial protein fraction. The arrow indicates the specific FLAG-Cas9 band. M1 – iBright™ Prestained Protein Ladder (#LC5615, Invitrogen™). M2 – PageRuler™ Prestained Protein Ladder (#26616, Invitrogen™). Saturated pixels are marked in red.

**
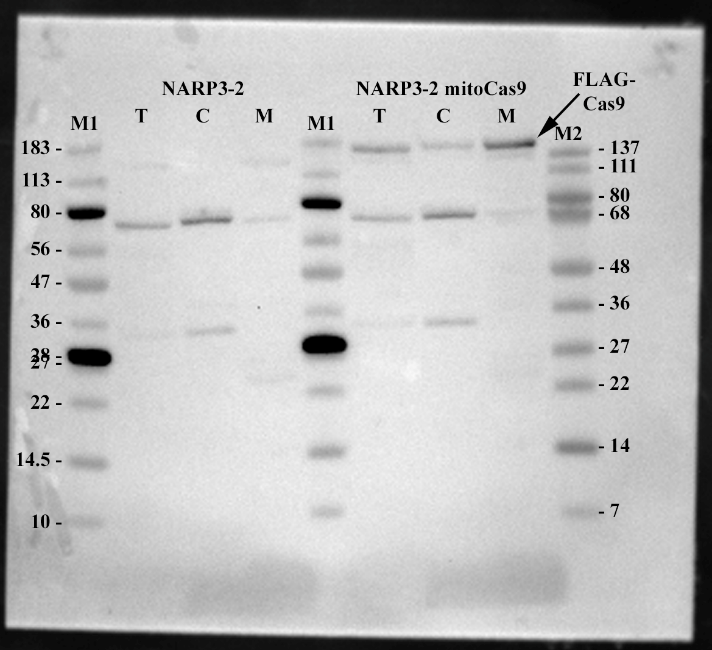

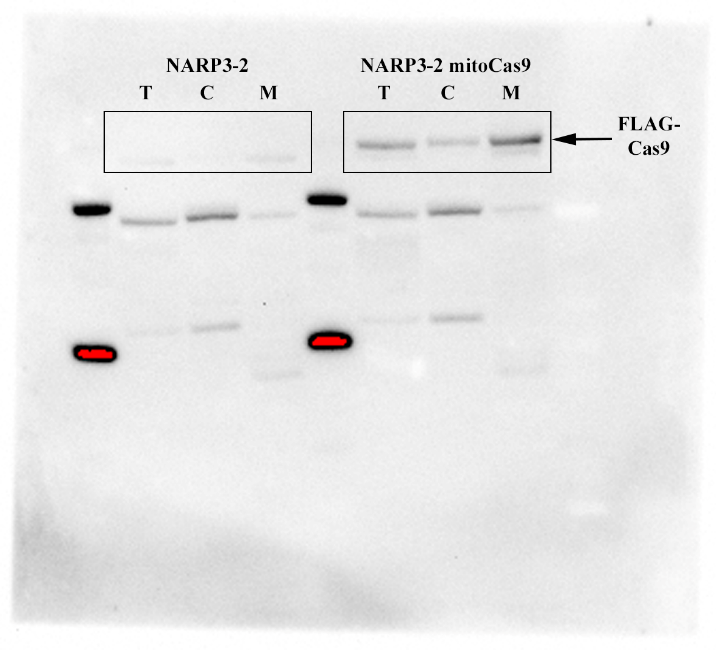
**


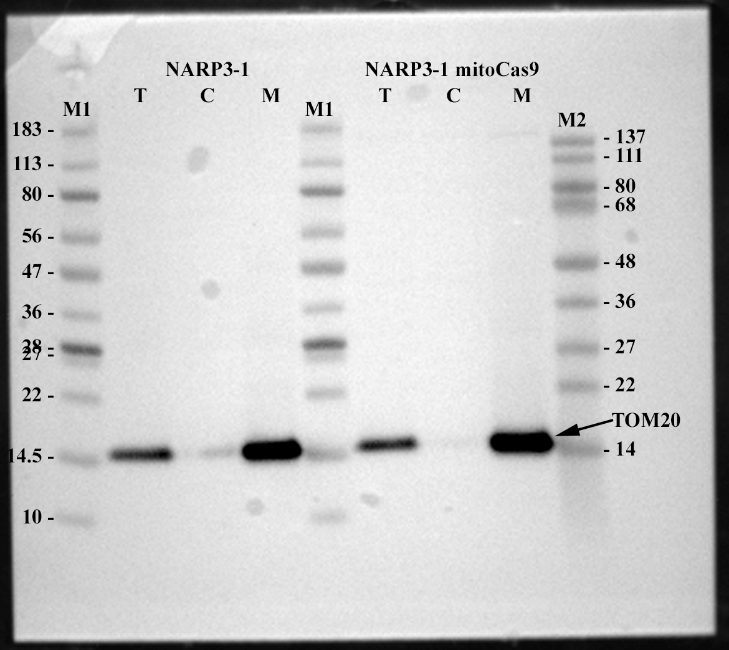
**3)** Protein expression of **TOM20** in parental **NARP3-1** and transgenic **NARP3-1 mitoCas9** cell lines determined by Western blotting. Left image - chemiluminescence signals, right image – overlay of chemiluminescence signals with the black-and-white image of the membrane. T – total cell lysate, C – cytoplasmic protein fraction, M – mitochondrial protein fraction. The arrow indicates the specific FLAG-Cas9 band. M1 – iBright™ Prestained Protein Ladder (#LC5615, Invitrogen™). M2 – PageRuler™ Prestained Protein Ladder (#26616, Invitrogen™). Saturated pixels are marked in red.


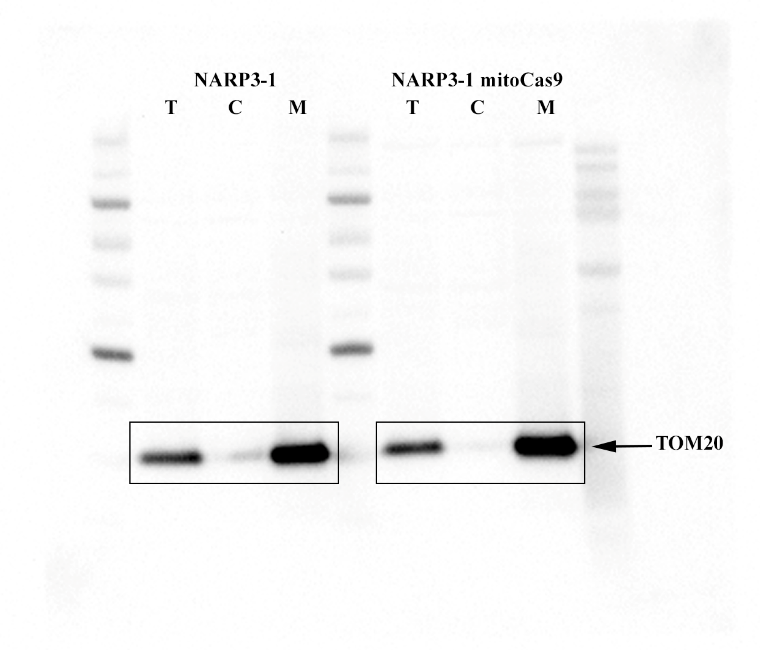


**4)** Protein expression of **TOM20** in parental **NARP3-2** and transgenic **NARP3-2 mitoCas9** cell lines determined by Western blotting. Left image - chemiluminescence signals, right image – overlay of chemiluminescence signals with the black-and-white image of the membrane. T – total cell lysate, C – cytoplasmic protein fraction, M – mitochondrial protein fraction. The arrow indicates the specific FLAG-Cas9 band. M1 – iBright™ Prestained Protein Ladder (#LC5615, Invitrogen™). M2 – PageRuler™ Prestained Protein Ladder (#26616, Invitrogen™). Saturated pixels are marked in red.


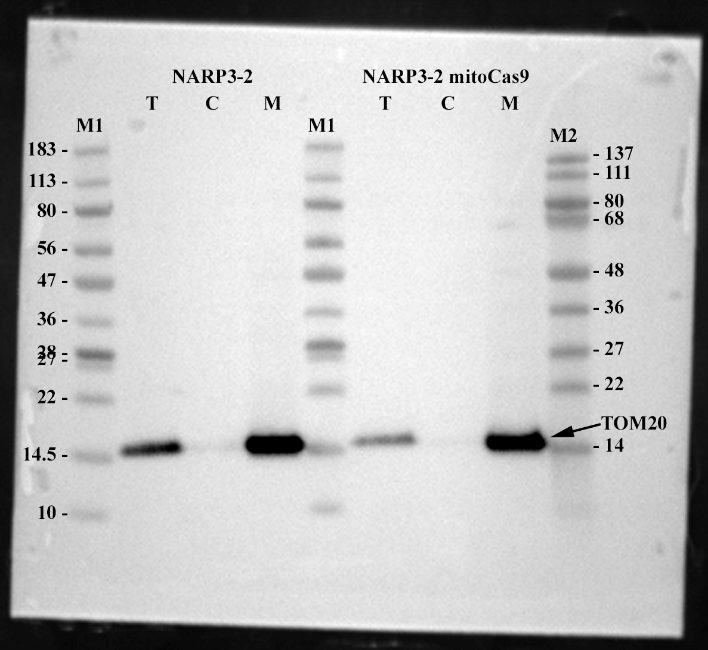

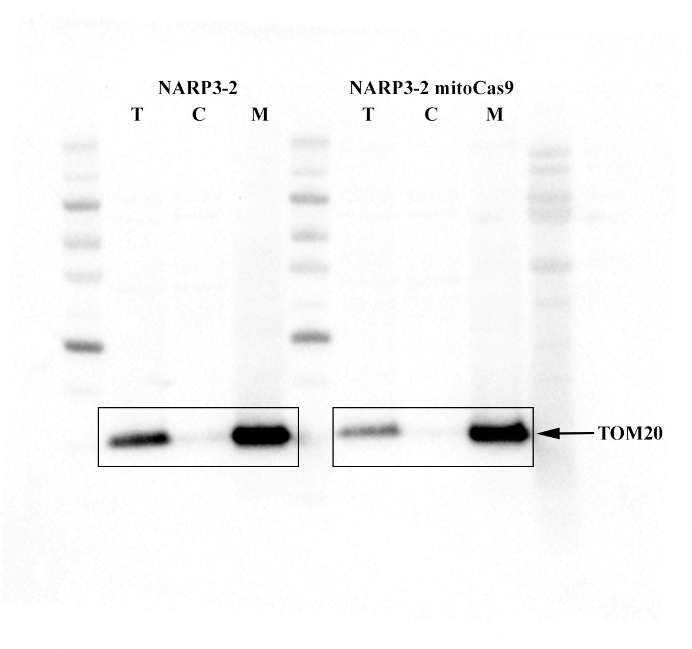


**5)** Protein expression of **GAPDH** in parental **NARP3-1** and transgenic **NARP3-1 mitoCas9** cell lines determined by Western blotting. Left image - chemiluminescence signals, right image – overlay of chemiluminescence signals with the black-and-white image of the membrane. T – total cell lysate, C – cytoplasmic protein fraction, M – mitochondrial protein fraction. The arrow indicates the specific FLAG-Cas9 band. M1 – iBright™ Prestained Protein Ladder (#LC5615, Invitrogen™). M2 – PageRuler™ Prestained Protein Ladder (#26616, Invitrogen™). Saturated pixels are marked in red.


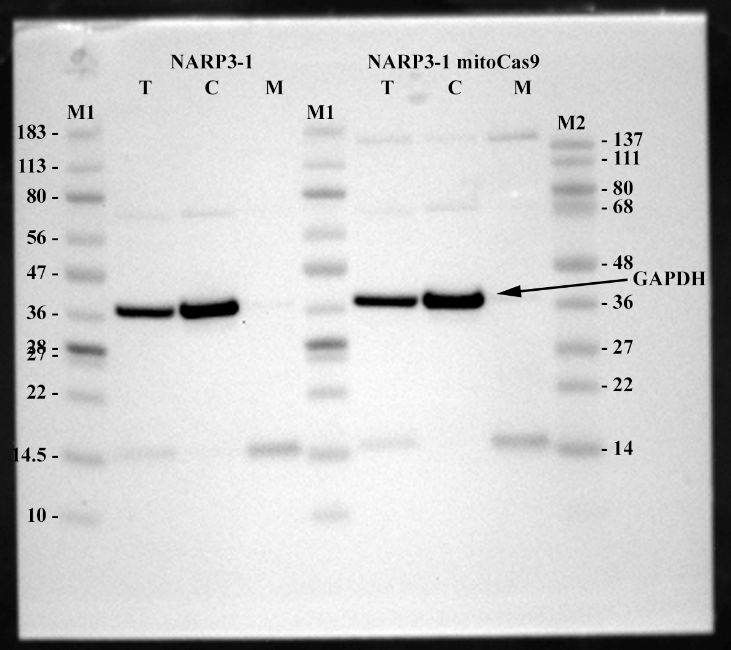

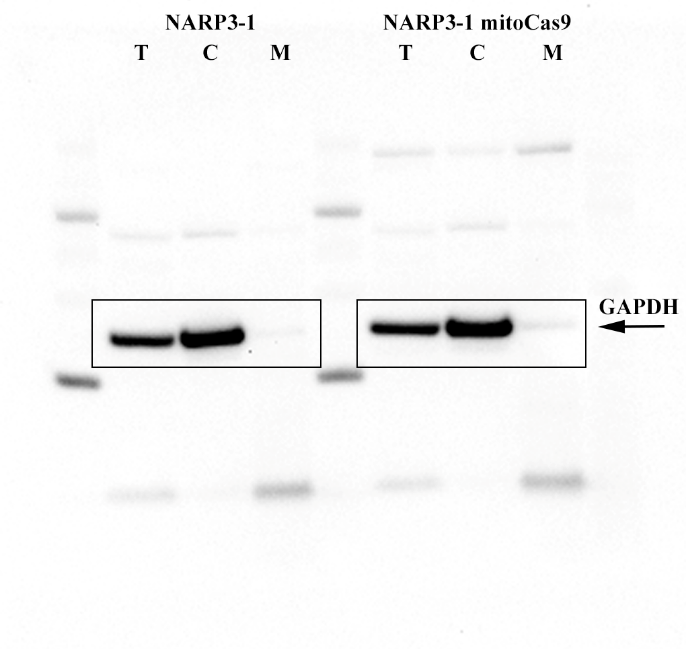


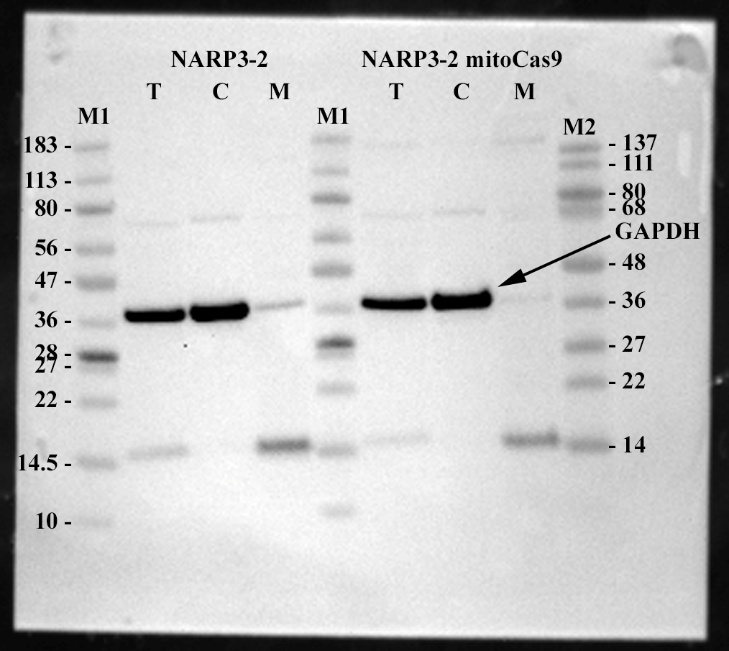
**6)** Protein expression of **GAPDH** in parental **NARP3-2** and transgenic **NARP3-2 mitoCas9** cell lines determined by Western blotting. Left image - chemiluminescence signals, right image – overlay of chemiluminescence signals with the black-and-white image of the membrane. T – total cell lysate, C – cytoplasmic protein fraction, M – mitochondrial protein fraction. The arrow indicates the specific FLAG-Cas9 band. M1 – iBright™ Prestained Protein Ladder (#LC5615, Invitrogen™). M2 – PageRuler™ Prestained Protein Ladder (#26616, Invitrogen™). Saturated pixels are marked in red.


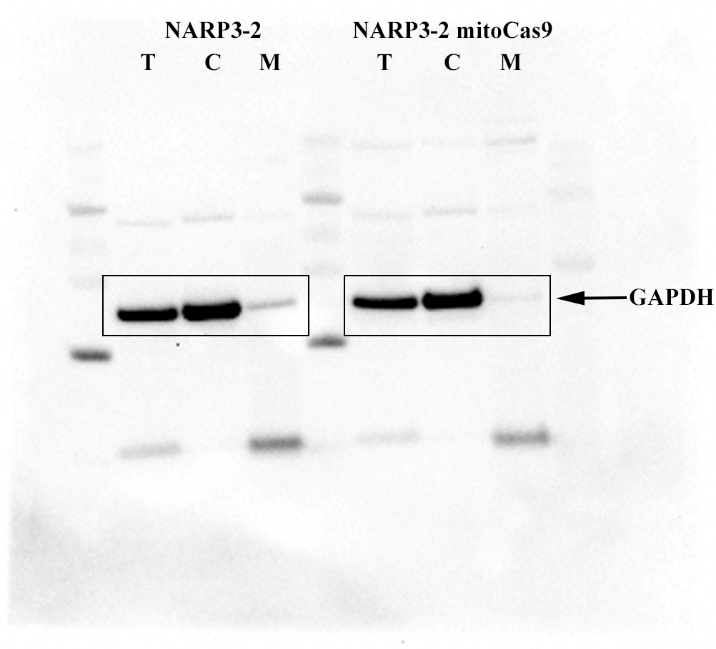


**Supplementary Figure S4.** Negative controls for mitoCas9 localization in the NARP3-1 and NARP3-2 cybrid cell lines using an anti-FLAG antibody. Immunofluorescent staining of the NARP3-1 **(A)** and NARP3-2 **(B)** cybrid cell lines. When the anti-FLAG antibody was used, a weak nonspecific signal uniformly distributed throughout the cytoplasm was detected (green signal). Mitochondria were labeled by expression of mitochondrially targeted TagRFP (red signal). DAPI was used for nuclear staining (blue signal). Scale bar, 10 µm.


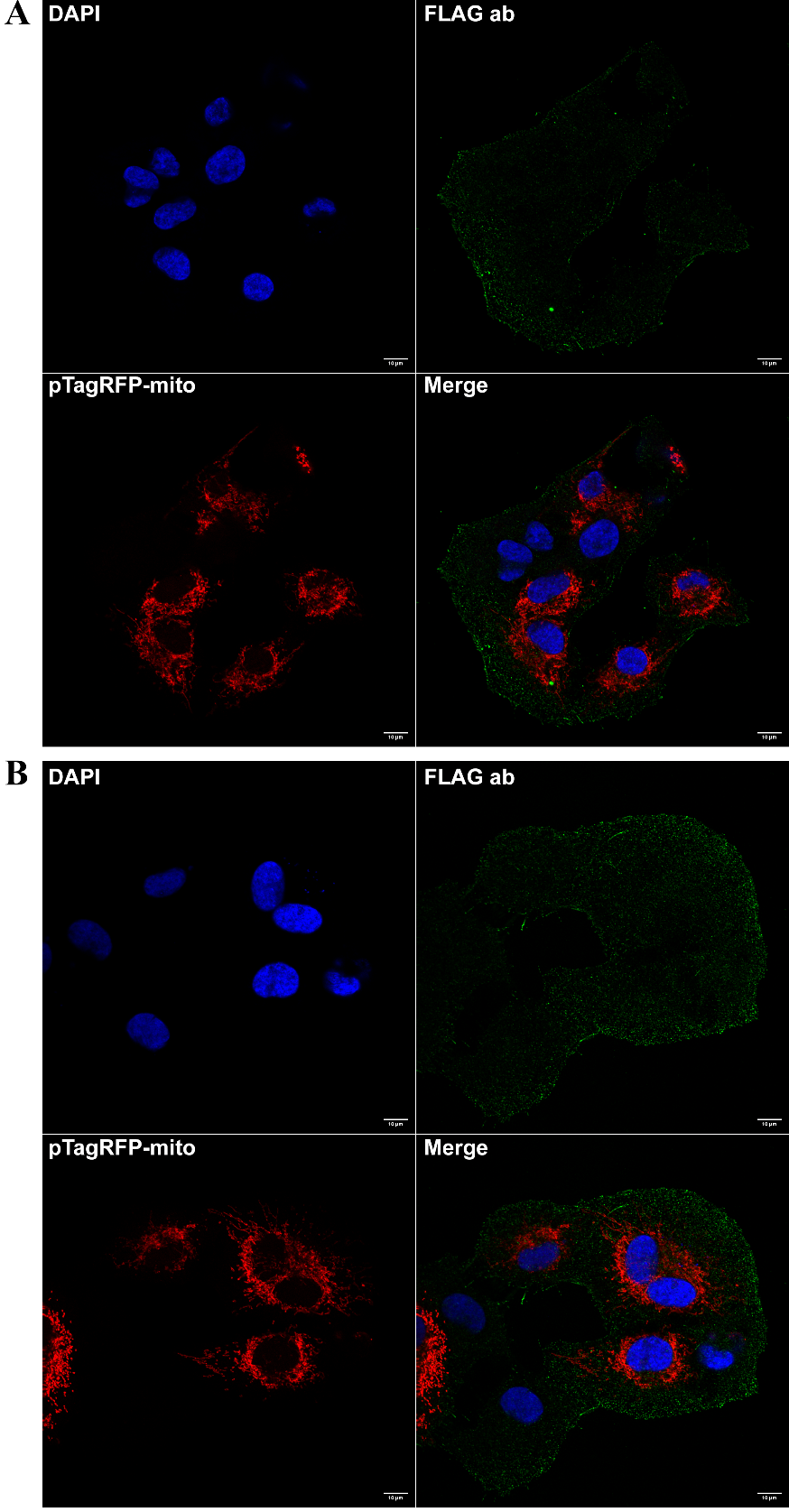


**Supplementary Figure S5: Original full-length gel images used in Figure 6B for the evaluation of mtDNA heteroplasmy level shift with irrelevant lanes crossed out.** PCR-RFLP analysis of mtDNA heteroplasmy levels after 2 and 6 days of treatment in NARP cybrid cell lines in vitro. Total DNA isolated from cells on days 2 and 6 post-treatment was amplified by PCR. The presence of 960 bp and 345 bp bands indicates the presence of the copies of mtDNA carrying the m.8993T>G mutation site recognised by the AvaI restriction enzyme. Untreated – cells without any treatment. m.8993T>G NEG – cells treated with gRNA targeting the m.8993T>G mutation but lacking the RNA import determinant (NEG). m.8993T>G RP-SLO — cells treated with the mitoCRISPR-Cas9 system containing RP-SLO gRNA. As a positive control, DNA was extracted from cells transfected with plasmids encoding components of the mtZFN system, which specifically target the m.8993T>G mutation. DNA isolated from the 2SA cell line, which does not contain the m.8993T>G mutation, was used as a negative control. M represents a 100 bp Plus Ladder (SibEnzyme). Data are presented as three biological replicates (n = 3), each analyzed in three technical triplicates (given in brackets).

**1)** **NARP3-1, day 2**


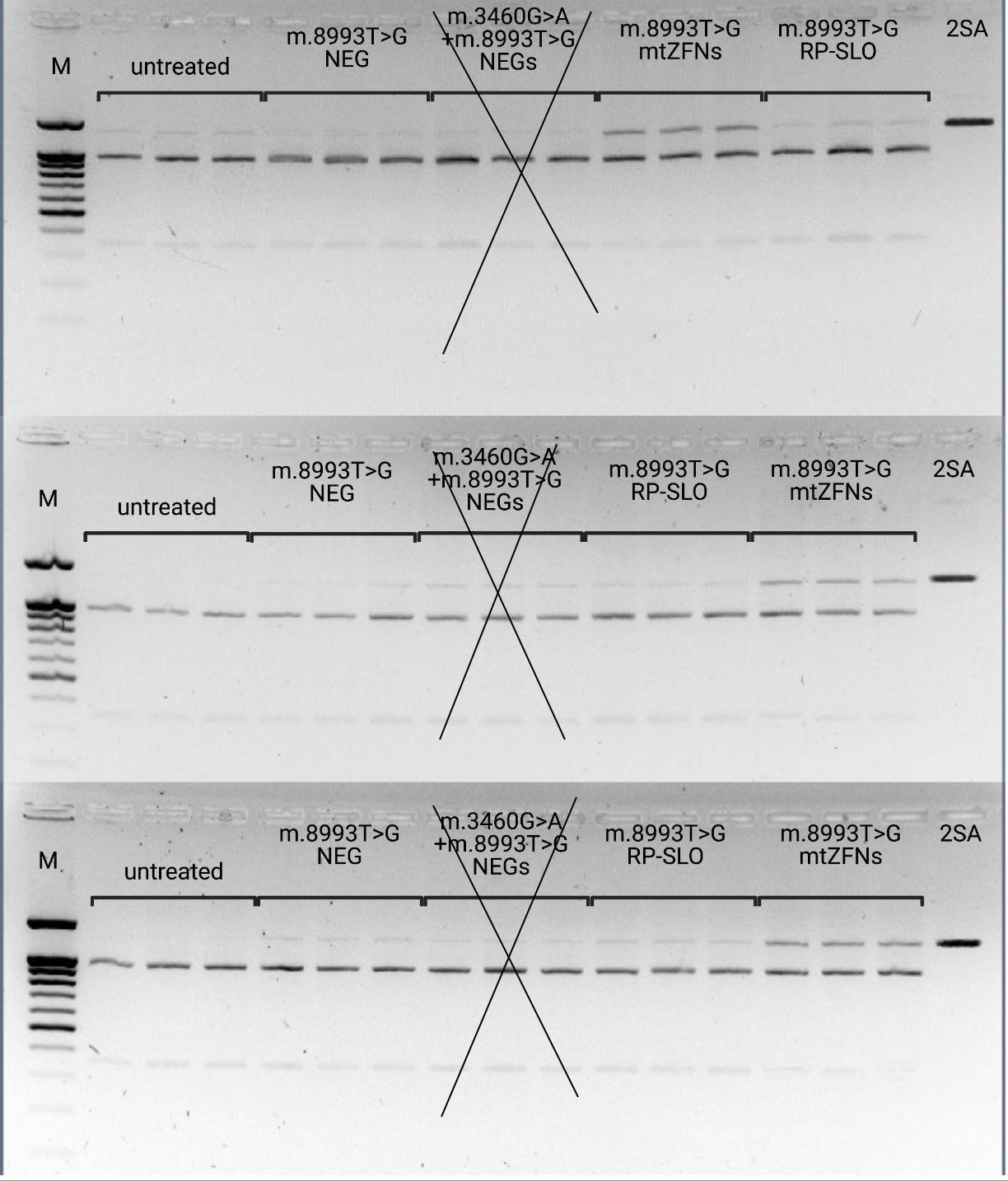


**2)** **NARP3-1, day 6**


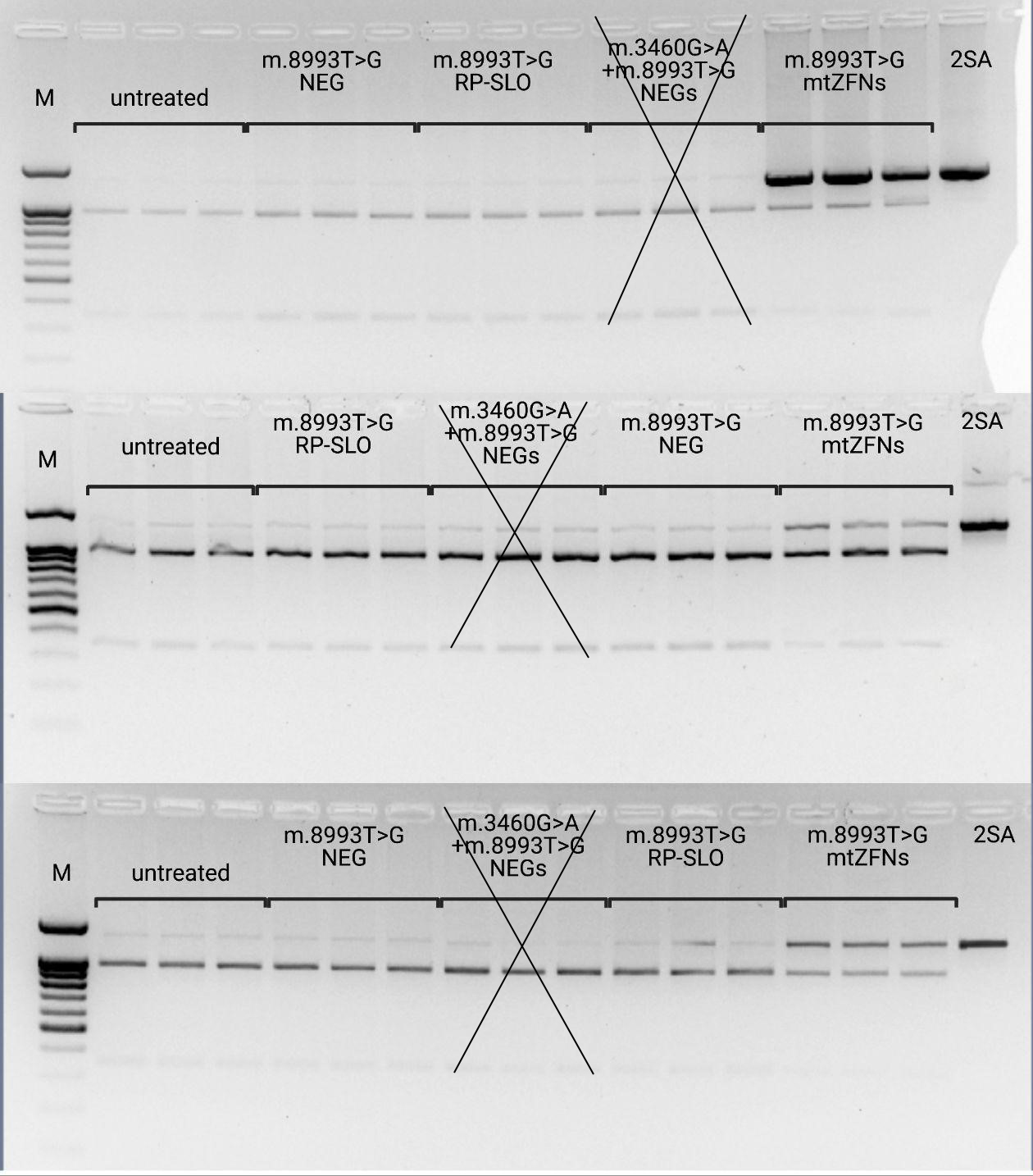


**3)** **NARP3-1 mitoCas9, day 2**


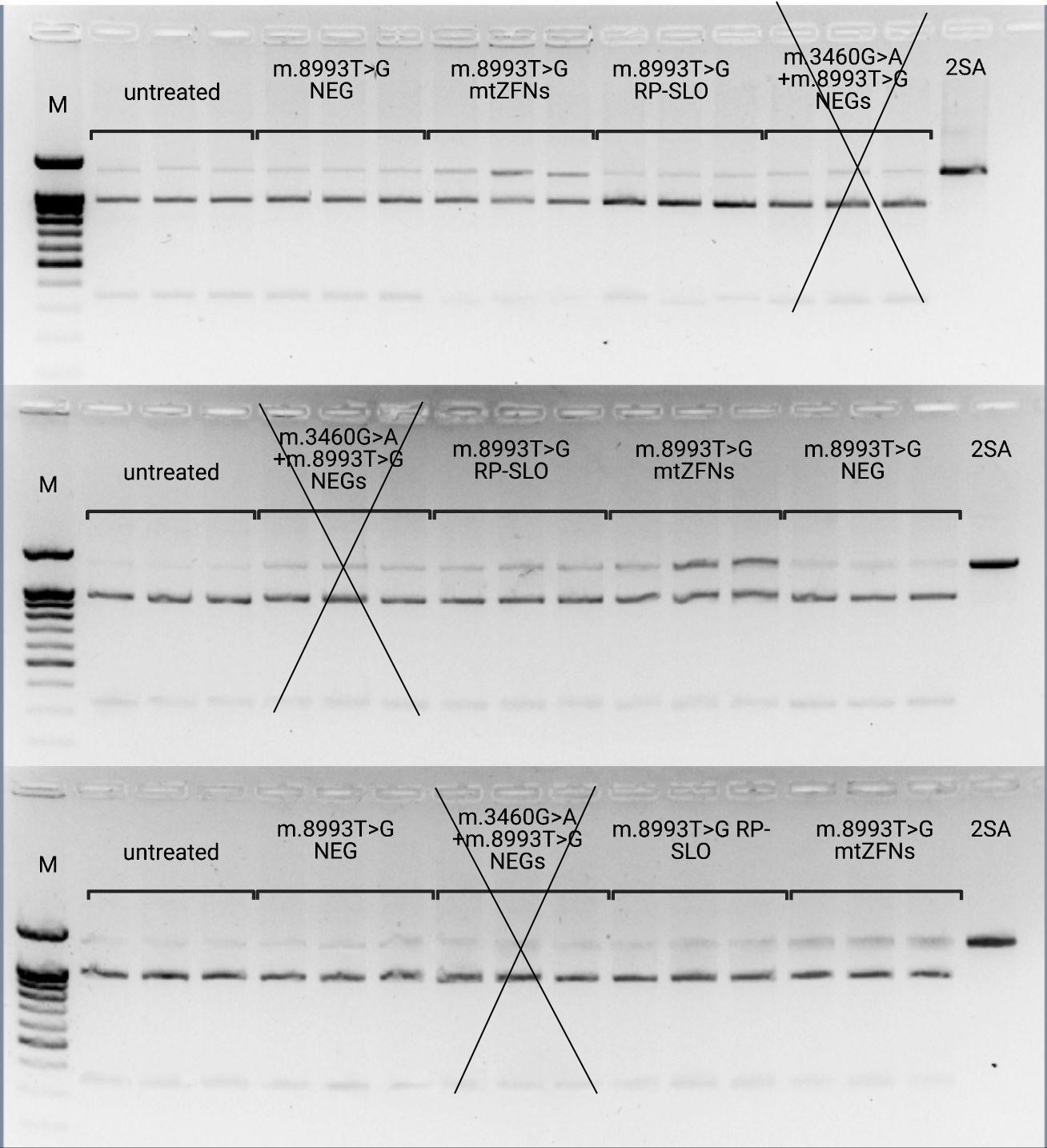


**4)** **NARP3-1 mitoCas9, day 6**


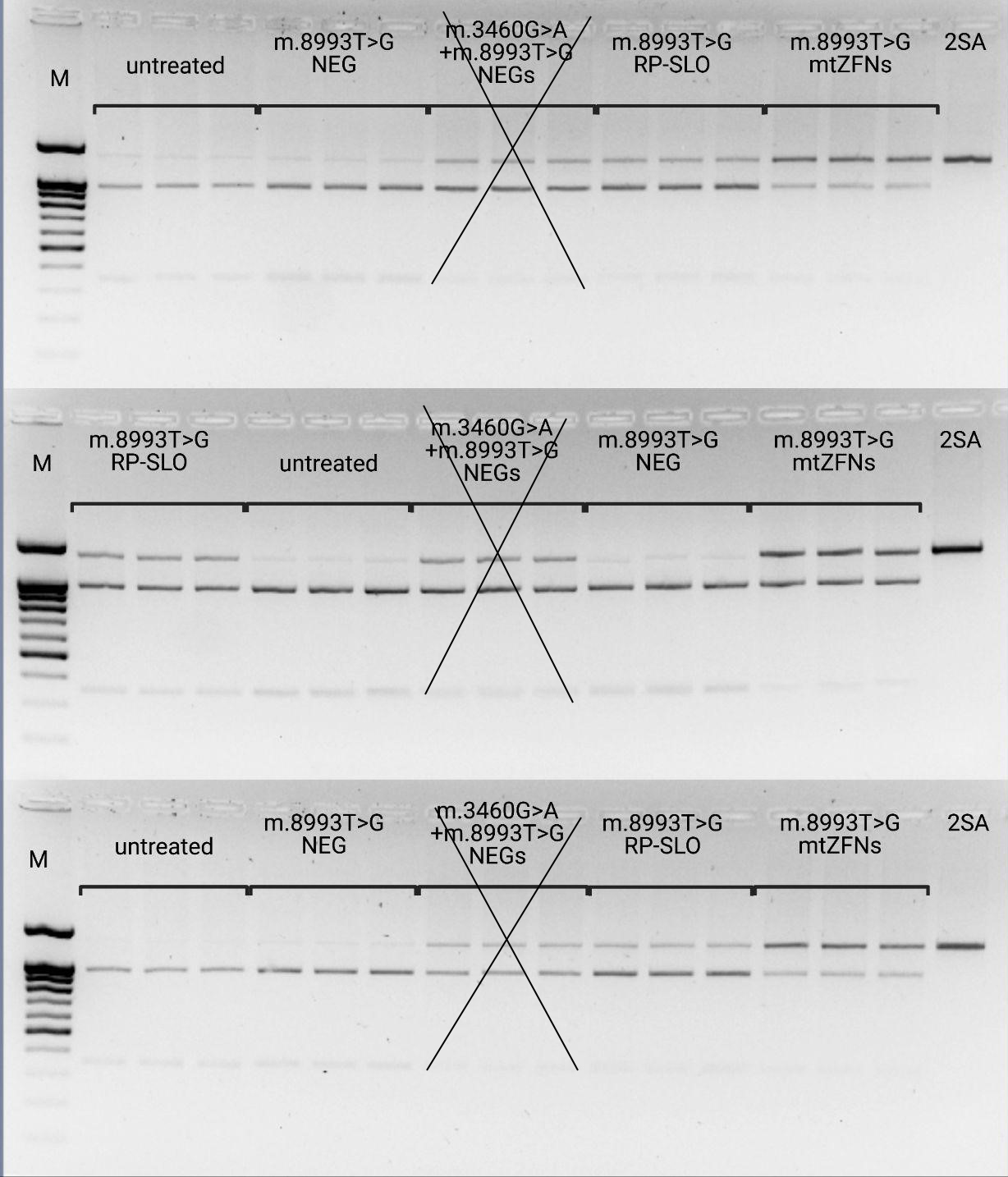


**5)** **NARP3-2, day 2**


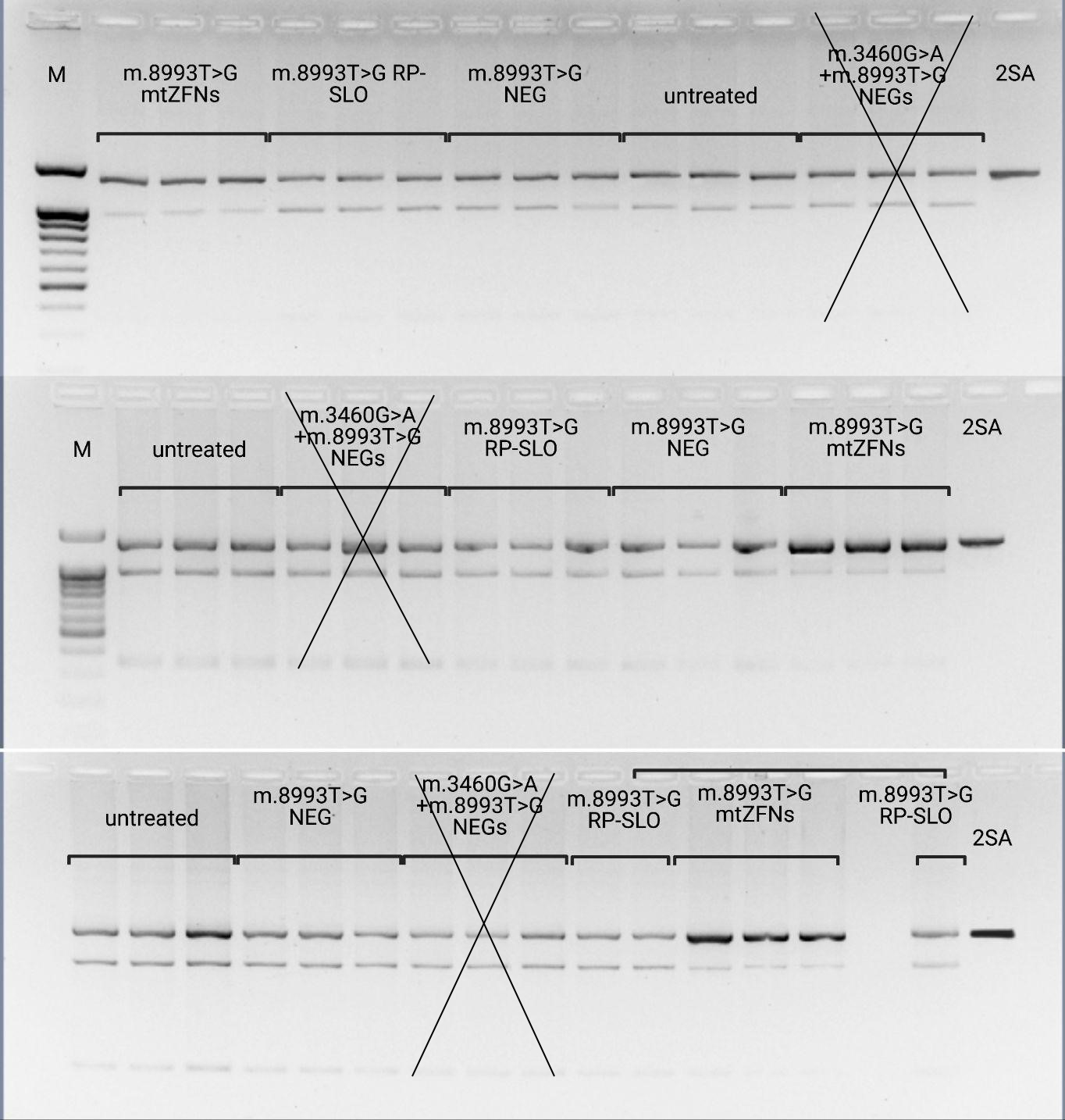


**6)** **NARP3-1, day 6**


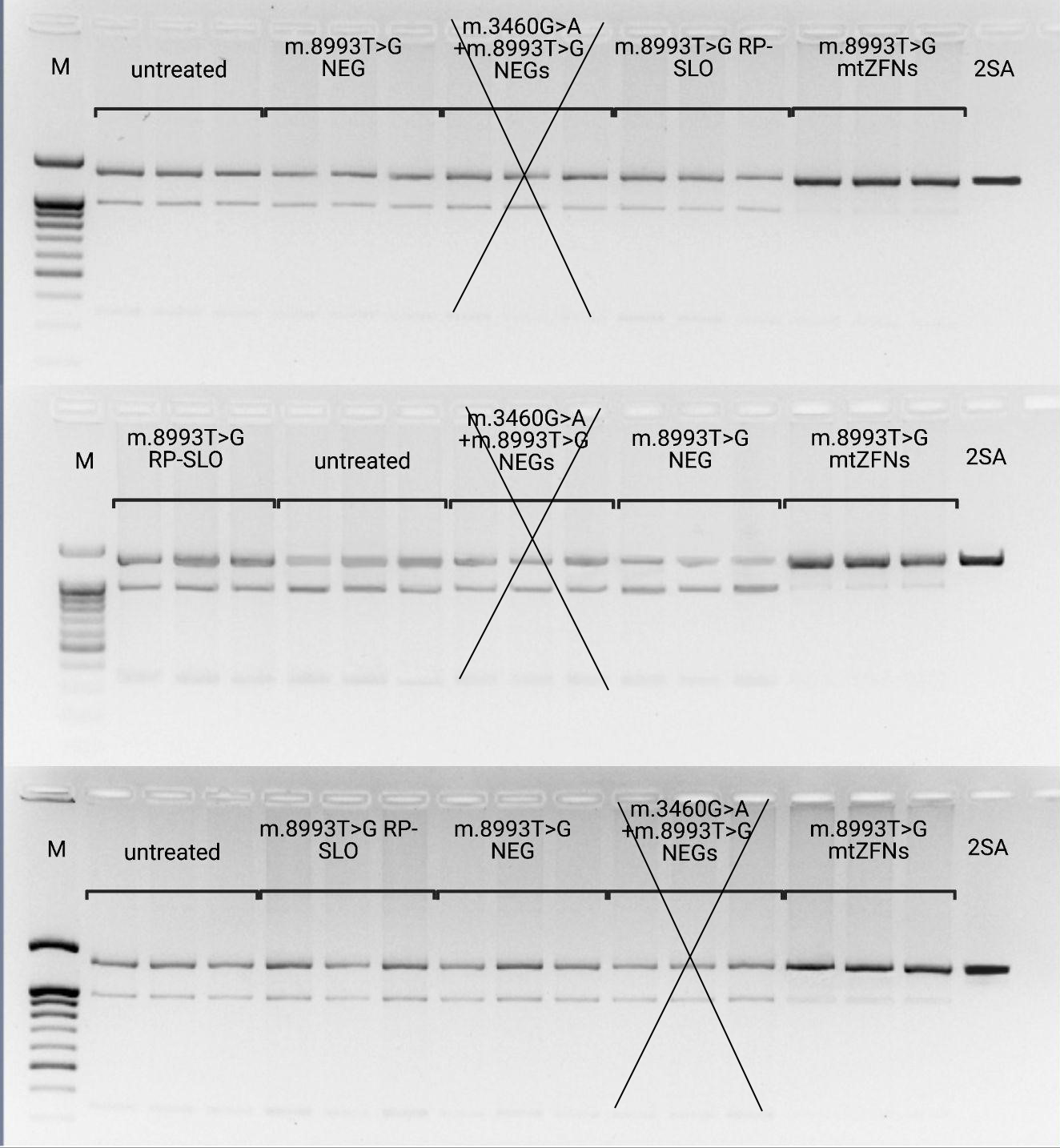


**7)** **NARP3-2 mitoCas9, day 2**


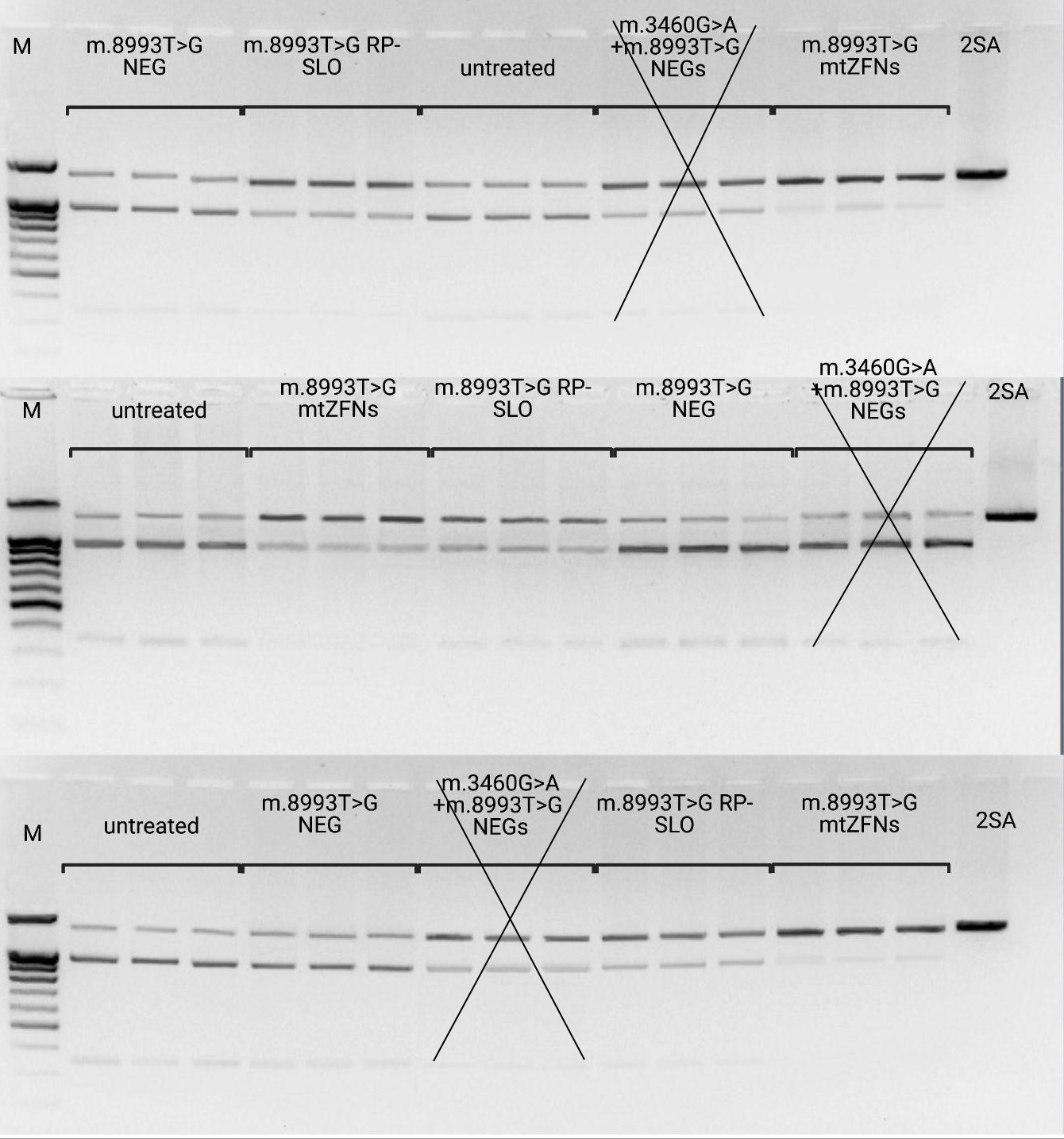


**8)** **NARP3-2 mitoCas9, day 6**


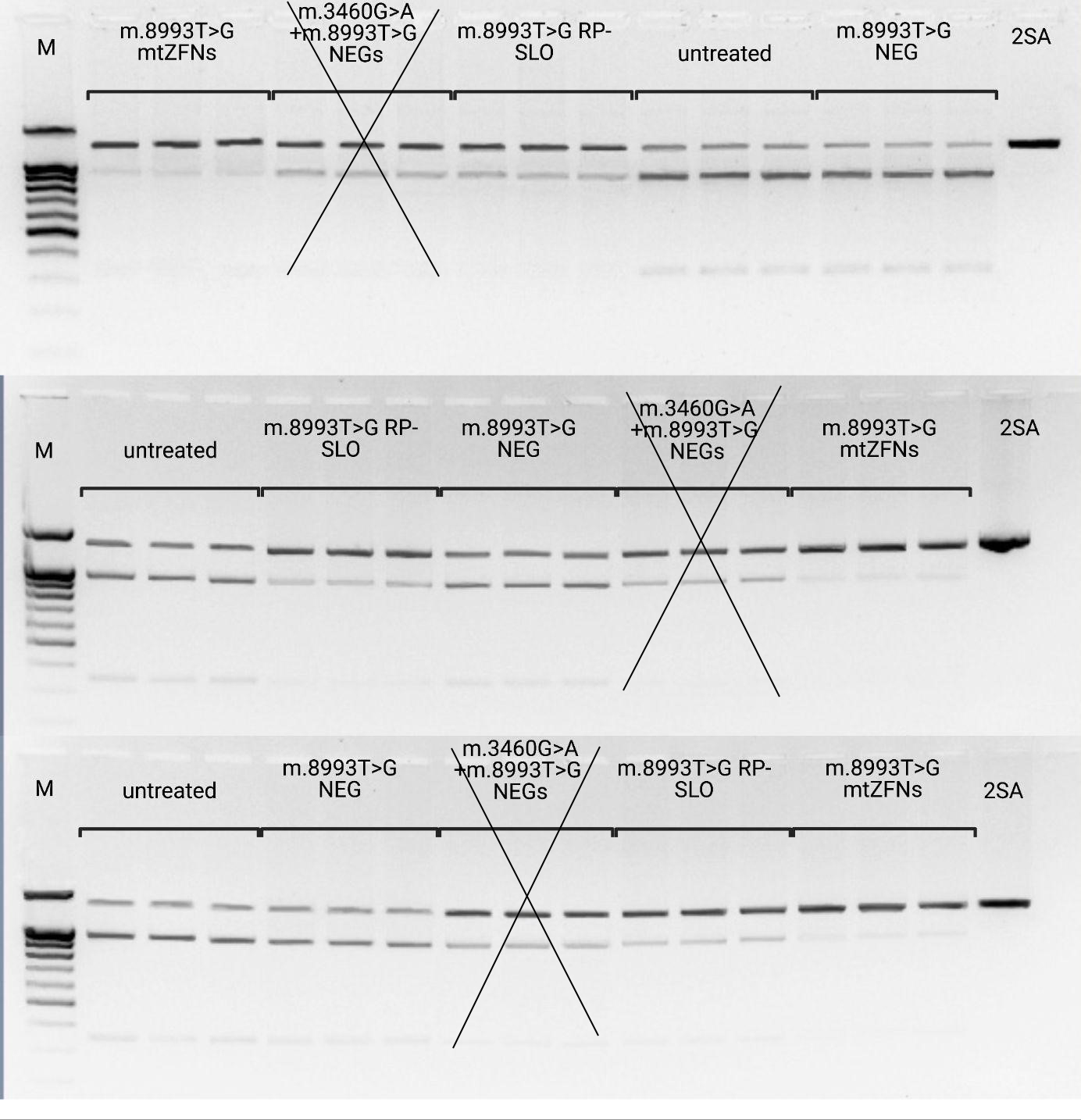

Supplement: Supplementary file 1 — Supplementary Material 1 [file 41598_2026_49007_MOESM1_ESM.docx]
