# Supplementary material for: Specific elimination of m.8993T>G mitochondrial haplotype in NARP cybrid cells by CRISPR-Cas9 system
Source: Sci Rep. 2026 Apr 28;16:19745. doi: 10.1038/s41598-026-49007-y (PMC13316044; doi:10.1038/s41598-026-49007-y)
Supplement: Supplementary file 2 — Supplementary Material 2 [file 41598_2026_49007_MOESM2_ESM.docx]

Supplementary Table 1. Efficiency of mitoCas9 expression in the NARP3-1 mitoCas9 and NARP3-2 mitoCas9 cell lines

| Cell Line | % of GFP-Positive Cells | Geometric Mean of GFP Fluorescence Intensity | Gene Copy Number of mitoCas9 |
| --- | --- | --- | --- |
| NARP3-1 mitoCas9 | 99,2 | 1692 | 25 |
| NARP3-2 mitoCas9 | 97,4 | 439 | 14 |

**Supplementary Table 2. All primer sequences used in this paper**

| **Primer Name** | **Sequence (5' to 3')** | **Application** |
| --- | --- | --- |
| H9931 | AACCACATCTACAAAATGCC | Screening of m.8993T>G mutation by PCR-RFLP and amplification of templates for *in vitro* transcription |
| L8305 | CCCCTCTACCCCCTCTAGAGCCCACTGTAAAGC |  |
| P24 | CCGACTAATCACCACCCAAC |  |
| mitoCas9-ddF | AAGAACGGCCTGTTTGGTAA | ddPCR amplification of the *mitoCas9* gene |
| mitoCas9-ddR | GTTGAAGCTTGGCATCTTCG |  |
| mitoCas9 probe | 6-FAM-GCCCTGTCACTCGGGCTGACCCC-BHQ-1 | ddPCR detection probe for the *mitoCas9* gene |
| PPR30-ddF | GATTTGGACCTGCGAGCG | ddPCR amplification of the *PPR30* gene |
| PPR30-ddR | GCGGCTGTCTCCACAAGT |  |
| PPR30 probe | HEX- TCTGACCTGAAGGCTCTGCGCG-BHQ-1 | ddPCR detection probe for the *PPR30* gene |
| B2M qF | TGCTGTCTCCATGTTTGATGTATCT | RT-qPCR amplification of the *B2M* reference gene |
| B2M qR | TCTCTGCTCCCCACCTCTAAGT |  |
| D-loop qF | CCTAACACCAGCCTAACCAGATTTC | RT-qPCR amplification of the mitochondrial D-loop region |
| D-loop qR | AGATTAGTAGTATGGGAGTGGGAGG |  |

**Supplementary Table 3. Comparison of CRISPR-Cas-based systems for mtDNA editing in human cells**

| **Study** | **Cas9 system** | **Guide RNA modification** | **Validation of mitochondrial localization** | **Observed effect** |
| --- | --- | --- | --- | --- |
| This study | SpCas9 + COX8A MTS + SOD2 3'-UTR | sgRNA + RP-SLO motif | Cas9: immunofluorescence + fractionation; gRNA: HF-TLR construct (fluorescence); RP-SLO indirectly via heteroplasmy shift | Heteroplasmy shift m.8993T>G ~16–20%; no respiratory improvement |
| Bi et al. (2022) – The Innovation (Bi et al. 2022) | SpCas9 + COX8A MTS + SOD2 3'-UTR | Unmodified sgRNA | Cas9: protease protection; gRNA: inferred via knock-in outcome | Knock-in efficiency 0.03–0.23% of mtDNA copies |
| Hussain et al. (2021) – Front Genet (Hussain et al. 2021) | SpCas9 + MLS1+MLS2 | sgRNA + RP-loop (5’ end) | gRNA: qPCR of mitochondrial fractions; Cas9 not shown | ND4 transcript and mtDNA reduced in mutant-targeting conditions |
| Wang et al. (2021) – Sci China Life Sci (Wang et al. 2021) | SaCas9 + COX8A MTS | Unmodified sgRNA | No direct RNA localization data; functional impact via InDel detection | Short InDels (2–25 bp) at microhomologous regions |
| Nikitchina et al. (2025) – CRISPR/Cas12a system (Nikitchina et al. 2025) | AsCas12a + COX8A MTS | Unmodified crRNA (~40 nt) | Functional validation via mtDNA deletion | Efficient site-specific deletions; no gRNA engineering required |
| Loutre et al. (2018) – IUBMB Life (Loutre et al. 2018b) | SpCas9 + COX8A MTS | sgRNA with hairpin RNA motifs | mtDNA depletion assay; no direct imaging | 2–3× mtDNA depletion only with dual sgRNAs |
| Schmiderer et al. (2022) – Sci Rep (Schmiderer et al. 2022) | SpCas9 + MLS3 + UTR1 | sgRNA + RP-loop (5’ or 3’) | mtDNA depletion-based assay | Strong depletion only with co-delivered sgRNA + mitoCas9 |
